# Supplementary material for: Local CO2 reservoir layer promotes rapid and selective electrochemical CO2 reduction
Source: Nat Commun. 2024 Apr 22;15:3397. doi: 10.1038/s41467-024-47498-9 (PMC11035706; doi:10.1038/s41467-024-47498-9)
Supplement: Supplementary file 1 — Supplementary Information [file 41467_2024_47498_MOESM1_ESM.pdf]

Supplementary Information

Local CO_2 Reservoir Layer Promotes Rapid and Selective Electrochemical CO_2 Reduction

Subhabrata Mukhopadhyay[1], Muhammad Saad Naeem[2,3], G. Shiva Shanker[1], Arnab Ghatak[1], Alagar R. Kottaichamy[1], Ran Shimoni[1], Liat Avram[4], Itamar Liberman[1], Rotem Balilty[1], Raya Ifraemov[1], Illya Rozenberg[1], Menny Shalom[1], Nuria López[2]*, Idan Hod[1]*

1- Department of Chemistry and Ilse Katz Institute for Nanoscale Science and Technology, Ben-Gurion University of the Negev, Beer-Sheva, 8410501, Israel.
2- Institute of Chemical Research of Catalonia (ICIQ-CERCA), The Barcelona Institute of Science and Technology (BIST) 43007 Tarragona, Spain.
3- Universitat Rovira i Virgili, Pl. Imperial Tarraco 1, 43005 Tarragona, Spain.
4- Department of Chemical Research Support Weizmann Institute of Science, Rehovot, 7610001, Israel.

Details	Page No
**Section S1.**<br>Table S1.<br>Figure S1.<br>Quantifying the density of missing linker defect in UiO-66-A-C membranes and loading of BA−CN groups in UiO-66-CN with the help of ICP-OES and 1H-NMR.<br>Electrochemical CO_2 reduction performance of high surface-area p-Bi: with, and without MOF membrane<br>Figure S2. Illustration of the ATR-IRRAS in Otto configuration experimental set-up<br>A note on the ATR-IRRAS spectroscopic set-up.<br>A note on the operando ATR-IRRAS spectroscopy<br>Quantification of solubilized CO_2 per node for Bi-UiO-66-B-CN under CO_2 purging conditions.<br>Reaction Mechanism by theoretical calculations.<br>Table S2-S7.	**S3- S11**
**Section S2.**<br>Figures S3-S37 Table S8-S11 and discussion on:<br>Use of −CN stretching peak as a probe to support the existence of interaction between −CN and *OCHO intermediate and between −CN and CO_2.<br>Use of quinone as redox probe to electrochemically determine CO_2 solubility for UiO-66-CN.<br>Necessary outcome about the intermediate *OCHO stabilization obtained from the isotope labelling experiments.<br>Electrochemical surface area calculation for different electrodes used in conventional H-cell.	**S12- S38**
**References**	**S41-42**

Section S1.

Table S1. Details of synthetic conditions for the preparation of Bi-UiO-66-A-C membranes.

Sample code	Amount of precursor solution	Time required to form the membrane
Bi-UiO-66-A	100 μL	2 hours
Bi-UiO-66-B	150 μL	4 hours
Bi-UiO-66-C	200 μL	6 hours

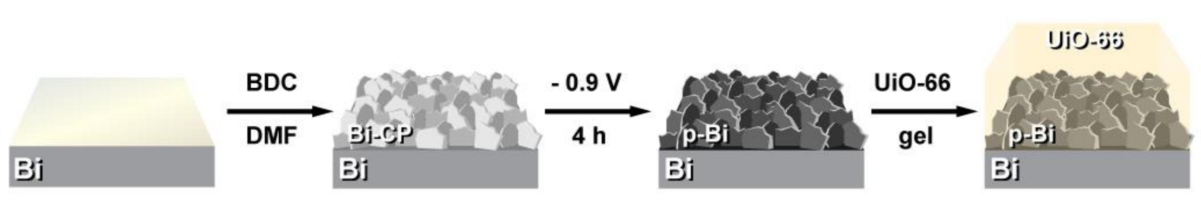

Figure S1. Schematic of synthesis of p-Bi-UiO-66 starting from Bi plate. First the Bi-based coordination polymer was prepared (Bi-CP), which was electrochemically converted to p-Bi before growing the MOF membrane atop it.

Quantifying the density of missing linker defect in UiO-66-A-C membranes and loading of BA−CN groups in UiO-66-CN with the help of ICP-OES and 1H-NMR.

Determination of density of Zr_6 nodes in Bi-UiO-66-(A-C) and Bi-UiO-66-B-CN

The density of Zr_6 nodes for Bi-UiO-66-A-C should be the same as that of UiO-66 gel. Thus, for ICP-OES analysis, a known amount of UiO-66 gel was digested in 5 ml of concentrated HNO_3 at 150 °C for 12 hours. 1 ml of the acid mixture was diluted to 10 ml by adding water and analyzed by ICP-OES.

From the experimentally obtained concentration of Zr ions in the 10 ml solution, the concentration of Zr_6 nodes was calculated.

The molecular formula of UiO-66 is $Zr_6O_4(OH)_4(OOC\text{-}C_6H_4\text{-}COO)_6$; molar mass 1664.06 g/mole

The density of Zr in UiO-66 was analyzed from ICP to be = 10.654 mg/L.

3.3 mg of UiO-66 contains 0.5326 mg of Zr.

Thus, 3.3 mg of UiO-66 contains 9.74 $\times 10^{-7}$ moles of Zr_6.

Thus, 2 mg of UiO-66 contains 5.9 $\times 10^{-7}$ moles of Zr_6.

Similarly, for UiO-66-CN, the density of Zr in UiO-66-CN was analyzed from ICP to be = 7.224 mg/L.

Thus, 2.5 mg of UiO-66 contains 0.3612 mg of Zr.

Thus, 2.5 mg of UiO-66-CN contains 6.61 $\times 10^{-7}$ mole of Zr_6.

Thus, 2 mg of UiO-66-CN contains 5.33 $\times 10^{-7}$ mole of Zr_6.

Now, the amount of BDC in 2 mg UiO-66 = 23.44 $\times 10^{-7}$ mole.

Considering the results obtained from ICP-OES analysis for UiO-66 gel, BDC/Zr_6 = 3.97.

Thus, the number of BDC per node = 7.94 ≈ 8 for UiO-66 gel.

So, number of defect sites per node = (12 – 8) = 4 for UiO-66 gel.

Amount of BDC in 2 mg UiO-66 = 29.9 $\times 10^{-7}$ mole.

Considering the results obtained from ICP-OES analysis for UiO-66-CN, BDC/Zr_6 = 5.6.

Thus, the number of BDC per node should be 11.2 for UiO-66 gel.

The excess BDC of (11.2 – 7.94) = 3.26 ≈ 3 per Zr_6-oxo node of UiO-66-CN is a result of the hydrolyzed BA-CN groups in addition to the already present BDC groups. The −CN group cannot withstand the high basic environment of 2 M NaOH, which was used to digest the MOF into an aqueous solution. The −CN groups get hydrolyzed to −COOH groups under the condition of digestive NMR sample preparation.

Thus, the loading level of BA-CN was ≈ 3 per Zr_6-oxo node.

Electrochemical CO_2 reduction performance of high surface-area p-Bi: with, and without MOF membrane.

The eCO_2RR for high surface area p-Bi was carried out in conventional H-cell set-up following the method dictated earlier for the electrochemical measurements performed in conventional H-cell. With the conversion of Bi-CP to p-Bi, a noticeable change in the surface (Figure S14, S15) can be observed. The gradual conversion of the Bi based coordination polymer to a mixed phase of bismuth and bismuth oxides could be confirmed by comparing the PXRD pattern (Figure S14) and DRIFTS-IR spectrum (Figure S14b) of Bi-CP with that of partially converted Bi-CP and the p-Bi.[1-3] The UiO-66-B-CN membrane was grown on top of p-Bi using UiO-66-B-CN gel (Figure S1). SEM-FIB analysis of the p-Bi-UiO-66-CN-B exhibits a continuous layer of UiO-66

membrane of ≈18 μm standing atop p-Bi of a thickness of ≈19 μm (Figure S15c). As the Bi^{3+} ions generated from the Bi-foil were the only source of Bi to form Bi-BDC coordination polymer, the bulk Bi was intricately connected to the p-Bi layer. p-Bi mainly consists of metallic Bi and Bi_2O_3 and trace amounts of BiO_2 as analysed by XRD (Figure S14a, Figure S15d).[1-3] A substantial increase in the surface area accompanied the formation of p-Bi. The electrochemically active surface area (EASA) of the p-Bi was over 18-times larger than Bi-foil (Figure S18, S19 and Table S9). Similarly, the overall catalytic current density was also ~15 times that of Bi-foil (Figure S17).

The FE_{HCOOH} for p-Bi gradually increased as potential was increased and reached a maximum of ≈50% at −0.85 V vs. RHE (Figure S16a) and then decreased to ≈30%. The p-Bi may consist of Bi nanoparticles under operational conditions, leading to its comparatively higher inherent HCOOH selectivity than Bi-foil. p-Bi-UiO-66-B showed an overall higher selectivity towards HCOOH formation, with the maximum FE_{HCOOH} of 72%. However, at potentials higher than −0.7 V, HCOOH selectivity starts decreasing, and at −0.9 V FE_{HCOOH} was only ≈40%. The effect of mass transport diffusion due to the UiO-66 membrane could increase the HCOOH selectivity but was not equally efficient at a high potential range. It might be the effect of significant depletion of local CO_2 concentration at high potentials. On the contrary, the FE_{HCOOH} for p-Bi-UiO-66-B-CN was higher than 80% throughout the potential range of −0.65 V to −0.9 V vs. RHE, while the highest FE_{HCOOH} was recorded to be 92 %. Such high values of FE_{HCOOH} at high potentials could be obtained because of the capacity of the nitrile (−CN) groups to (a) increase the local CO_2 concentration by a factor of ~27 compared to that of the bulk and (b) stabilize the CO_2 reduction intermediate. Furthermore, higher j_{HCOOH} for p-Bi-UiO-66-B-CN indicates a faster CO_2 reduction kinetics (Figure S16c). The increased local concentration of CO_2 near the Bi catalytic site can be the most crucial factor behind accelerated CO_2 reduction and suppressed HER (Figure S16 a-d). Thus, growing a thin-film UiO-66-CN membrane on top of a flat Bi-electrocatalyst and high surface area p-Bi is equally effective for CO_2 to HCOOH conversion.

We were interested to understand the long-term stability of the p-Bi-UiO-66-CN to realize the applicability of the MOF membrane approach. FE_{HCOOH} was maintained above 80% throughout a chronoamperometric measurement of 6 hours (Figure S20). Post-electrolytic characterization of the catalyst was performed by XRD, and SEM (Figure S21, S22). The structure of the Bi catalyst and the UiO-66-CN membrane were found to be unaltered by the bulk electrolysis. Thus, p-Bi-UiO-66-B-CN can be considered a robust and efficient catalytic system to synthesize HCOOH from CO_2 in an aqueous medium. At the same time, UiO-66-B-CN can function as a robust and efficient catalytically inactive MOF overlayer to modify the course of the reaction.

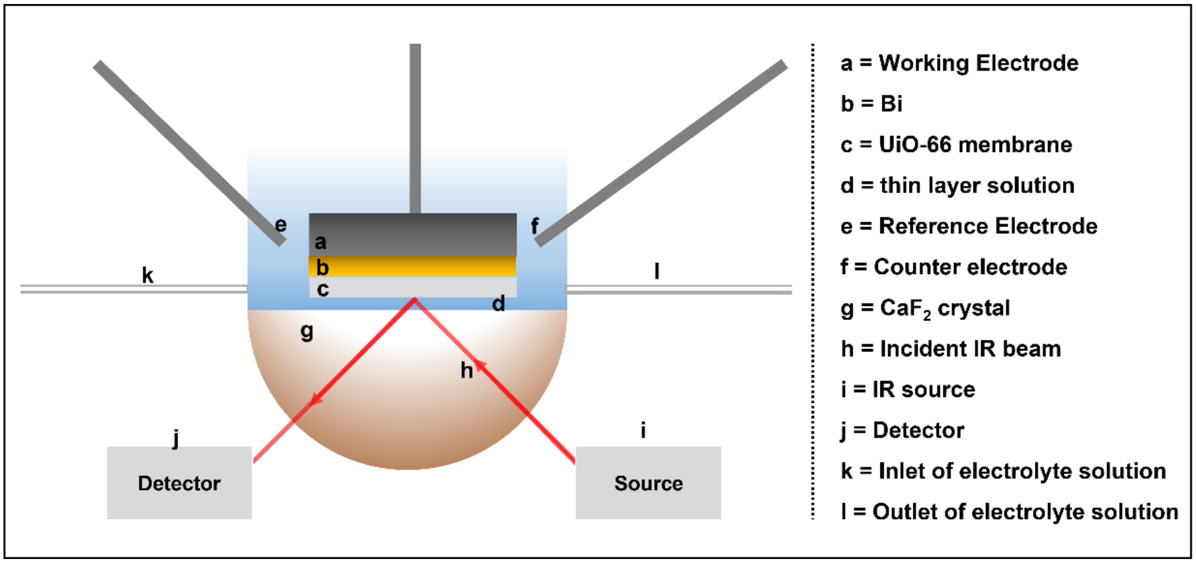

Figure S2. Illustration of the ATR-IRRAS in Otto configuration experimental set-up. The nature of b and c in the set-up varies for different measurements.

A note on the ATR-IRRAS spectroscopic set-up.

Please note that, in our study we have combination of ATR element with a thin layer cell, where we try to maintain the thickness of the solution layer so thin that the evanescent IR wave can penetrate through the solution layer and senses the species at the electrode surface. This technique ensures the increase in sensitivity (almost 5-fold in signal to noise ratio) compared to normal external reflection spectroscopy. Also, unlike external reflection method, the IRRAS does not suffer from background adsorption in the bulk of the electrolyte and therefore it has been reported to be used with particularly any reflecting surfaces providing the scope of a wide variety of spectroelectochemical studies.[4-6]

During the prolonged (more than 15 minutes) experiment of ATR-IRRAS measurement of analyte solution by purging CO_2 and Ar in cycles, the concentration of the atmospheric gas mixture (CO_2 and humidity) can vary. This may result into variation of the true background data at a given instance and lead to negative absorbance for contentious measurements. It is beyond the scope of the experiment to account for such variation. This is because collecting background between each measurement, would lead to overall misleading results, as here we are investigating the relative variation in the peaks of gaseous and dissolved CO_2. A similar negative absorbance was present, in low CO_2 concentration measurements for UiO-66 MOF (Figure 6a in main text). Importantly, it was absent for high CO_2 concentration measurements for UiO-66 MOF and for all measurements for UiO-66-CN (Figure 6b in main text). Since, we are not using this ATR-IRRAS data (negative peak at 2342 cm^{-1}) for quantitative measurements, it does not affect our data interpretation. Also, the

data is collected here by purging CO_2 and Ar in cycles, which causes rapid passage effect and accounts at least partly for the observed negative absorbance. However, often it has almost no physical significance and is a result of instrumental parameters and conditions during the data collection.[7-8]

A note on the operando ATR-IRRAS spectroscopy

There is indeed a chance that the thin layer configuration in ATR-IRRAS can affect the mass transport and thereby alter the course of an electrocatalysis while doing the operando experiments. Therefore, while doing the spectroelectrochemical measurements we always double checked the current density vs. time (j vs t) plots for all the three catalyst systems studied under the same condition. We always ensured a constant high flow of electrolyte solution (20 ml/min) and since a steady state catalytic current was achieved within a small time period, which was typically of the order of 60 second, for every experiment we waited for 60 second before collecting the data. This approach has helped us to minimize and normalize the mass-transport limitation for all the three catalytic systems which is shown in the mentioned j vs t plots (Figure S34), where the observed steady-state current density (after 60 seconds) for UiO-66-CN-MOF is greater than that of UiO-66 MOF, which in turn is greater than that bare Bi electrode under the same potential. Thus, we could safely hypothesize that the three catalyst systems analyzed by the spectroelectrochemical measurements resemble the corresponding catalyst systems studied in conventional H-cell and flow-cell set-up.

Another aspect of the data collection with spectroelectrochemical measurements is to be reminded of the probable role of the two important factors: (a) applied potential and (b) surface coverage for the observed peak positions in the spectroelectrochemical measurements.

The shift in band/peak position in operando ATR-IRRAS measurement, with varying the applied potential is known as the Stark effect. Stark shift is most observed for systems with polarizable probe moiety present within the dielectric layer.[9-11] Thus, among the three catalyst systems in our case i.e., only Bi, Bi-UiO-66 and Bi-UiO-66-CN, the Bi-UiO-66-CN is more prone to undergo Stark shift as it is the only system with a strongly polarizable probe -CN functionality. However, in our case the frequency shift with varying potential was found to be very less to almost negligible for the −CN probe moiety (at 2237 cm^{-1} in Figure 8d, main manuscript). Such negligible Stark shift can be the combined effect of the catalyst structures and the experimental set-up. As mentioned earlier, the probe moiety (in this case the −CN) must be present inside the dielectric layer to undergo Stark shift. In our case the dielectric layer is the aqueous double layer formed during the spectroelectrochemical

measurement. Now, Stark tuning rate depends on the Debye length of the aqueous double layer. Importantly, the Debye length is inversely proportional to the ionic strength of the electrolyte. For our spectroelectrochemical measurements, the ionic strength of 0.1M $NaHCO_3$ electrolyte solution is low. Thus, the expected Debye length should be extremely short, and the double layer should be tightly compressed. This results in a field which is supposed to be strong at the electrode surface and decays rapidly before reaching the probe molecule. Thus, no notable Stark shift was observed for the Bi-UiO-66-CN system. Hence, it can be said although nitriles can be used as a very good probe for vibrational stark effect owing to its larger dipole, in our experimental condition the electrical double layer decays before reaching the nitrile probe causing no significant vibrational frequency shift. Furthermore, the chances of notable vibrational Stark shift for the *OCHO intermediate is also low, since the dipole is considerably lower and the intermediate should be weakly bound to the catalyst surface

Likewise, the effect of surface coverage on the observed peak positions for the operando ATR-IRRAS measurements seems also negligible in our case. In general, increased surface coverage by a probe moiety can lead to interaction among them, causing a shift in peak position. As earlier mentioned, there are two potential probe moieties involved in our case, particularly for Bi-UiO-66-CN, which are the *OCHO and −CN. To account for the surface coverage of the −CN groups we need to look deeper into the structure of UiO-66 MOF. In the structure of UiO-66-CN, each of the Zr_6-oxo node is coordinated by nine BDC (benzene dicarboxylate) linkers and three deprotonated 4-cyanobenzoic acids (BA-CN). Considering the average structure, the three BA-CN should be well separated from each other in space and rigidly tethered to the Zr_6-oxo metal node. Thus, there is very low chance of the −CN groups of UiO-66-CN to interact with one another. Therefore, surface coverage effect of −CN on the shift of wavenumber in ATR-IRRAS measurement is not noticeable, even though the concentration of −CN is quite high near the electrode surface. On top of that, as *OCHO is being stabilized by the −CN, their surface coverage is as well determined by the MOF induced −CN spacing, thus are also unlikely to react with each other.

Quantification of solubilized CO_2 per node for Bi-UiO-66-B-CN under CO_2 purging conditions.

The loading level of BA-CN was ≈ 3 per Zr_6-oxo node.

For defective UiO-66-CN gel, the formula unit is $Zr_6C_{44}N_{1.5}O_{27}H_{24,}$ and so molar mass = 1553.09 g/mole.

Density of UiO-66 gel = 0.0108 g/ml.

Thus, the number of moles of UiO-66-CN in the UiO-66-B-CN membrane = 1.043 × 10^{-6} mole.
Now, the number of CO_2 molecules attached per node of Zr_6-oxo node = 1.47.
Thus, moles of CO_2 attached to 150 μl of UiO-66-B-CN membrane = 1.53 × 10^{-6}.
The geometrical area of the coated MOF membrane on the Bi foil under consideration is 1 $cm^{2,}$ and from FIB analysis of the thickness of the UiO-66-B membrane, it was found to have a thickness of ≈ 18.3 μM. Thus, the volume of the UiO-66-B-CN membrane should be ≈ 0.00144 cm^3

Thus, during the CO_2 purging cycle of the ATR-IRRAS measurements of CO_2 solubilization, when the solution is saturated with CO_2 by continuous purging, the UiO-66-B-CN membrane should contain 1.53 × 10^{-6} mole of CO_2 in excess to the bulk solution within its volume of 0.00144 cm^3 because of the −CN functionality.

Thus, Additional CO_2 concentration in Bi-UiO-66-B-CN = (1.53 × 1000/0.00183) × 10^{-6} ≈ 0.82 (M)

Reaction Mechanism by theoretical calculations.

The eCO_2RR mechanism can be thought through two different mechanisms (Figure 7 in the main manuscript). The first one encompasses CO_2 adsorption and activation (electron pulling from the surface) and a subsequent PCET step with the formation of surface adsorbed formate (*OCHO) that can then be dissolved.[12] The CO_2 activation is considered as an electron transfer process and hence is decoupled from the subsequent step to better understand the effect of the modified interface. The alternative is proton adsorption with the formation of hydride on the surface, through a Heyrovsky mechanism,[13] that is transferred to a CO_2 in the solution close to the surface.

The CO production entails CO_2 adsorption as *COO following a PCET step which leads to *COOH and finally another PCET step results in H_2O and CO.

Table S2. DFT C-O bond distance, O-C-O bond angle and CO_2 vibrational frequencies for CO_2 molecule in gas phase and different interfaces under study. A peak shift of 13 cm^{-1} and bond angle difference of 1.8° was observed between CO_2 in gas phase and CO_2 interacting with Bi-UiO-66-CN.

**Structure**	**Distance C-O bond in CO_2 (Å)**	**Angle O-C-O in CO_2 (°)**	**Frequency CO_2 (cm^{-1})**
CO_2 (gas phase)	1.18	180.0	2366.5
Bi (0001) + CO_2	1.18	179.2	2354.2
UiO-66-CN + CO_2	1.19	178.3	2367.0
Bi-UiO-66 + CO_2	1.17	179.3	2354.6
Bi-UiO-66-CN + CO_2	1.17	178.2	2353.0

Table S3. DFT *OCHO vibrational frequencies with Bi(0001) and Bi-UiO-66-CN interfaces. A peak shift of 22 cm^{-1} was observed.

**Structure**	**Frequency** *OCHO **(cm^{-1})**
Bi (0001)+ *OCHO	1579.4
Bi-UiO-66-CN + *OCHO	1557.3

Table S4. DFT vibrational frequencies for free −CN (Bi-UiO-66-CN), OCO interacted −CN, and *OCHO-interacted −CN, respectively. The effect of solvated electrolyte cation (Na^+) and the Stark effect (U= -0.6 V_{RHE}) were included to compute OCO interacted −CN, and *OCHO-interacted −CN (See method section in main text for details).

**Structure**	**Frequency (cm^{-1})**
* (free -CN)	2238.8
OCO interacted −CN	2242.0
*OCHO-interacted −CN	2280.2

Table S5. DFT vibrational frequencies for OCO interacted −CN, and *OCHO-interacted −CN, with and without the Stark effect (U=-0.6 V_{RHE}). See method section in main text for details.

**Structure**	**Frequency (cm^{-1}) no Stark effect**	**Frequency (cm^{-1}) with Stark effect at U = -0.6 V_{RHE}**
OCO interacted −CN	2248.1	2244.8
*OCHO-interacted −CN	2250.9	2252.1

Table S6. DFT vibrational frequencies for *OCHO on different interfaces with $^{12}CO_2$ and isotope labelled $^{13}CO_2$. See method section in main text for details.

**Structure**	**Frequency (cm^{-1}) using $^{12}CO_2$**	**Frequency (cm^{-1}) using $^{13}CO_2$**
Bi(0001)	1579.5	1540.4
Bi-UiO-66	1580.9	1541.0
Bi-UiO-66-CN	1557.3	1525.9

Table S7. Computational characterization of different Bi-UiO-66-CN models. In Zr-Bi (1) the excess UiO-66 oxygen atoms bound to Bi at different sites whereas the excess oxygen atoms are completely removed in Zr-Bi (2). The -CN frequencies, N bader charges and Bi 2s XPS shift (Bi atoms indicated with black) for all

structures largely remain unchanged for all Bi-UiO-66-CN models.

Model	Linkage	-CN Frequency (cm^{-1})	Bader Charge for N	Bi 2s XPS Shift (eV)	Bi-Zr Distance (Å)	Bi-N Distance (Å)	C-N Distance (Å)
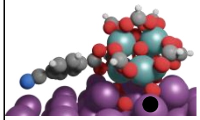	O-Bi	2238.7	-1.12	-0.17	4.19	3.66	1.17
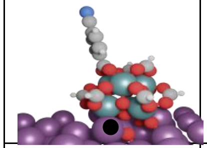	O-Bi$_{\perp}$	2238.7	-1.08	-0.14	4.13	14.87	1.17
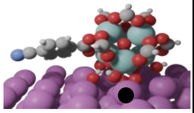	OH-Bi	2245.2	-1.07	-0.21	3.82	5.29	1.17
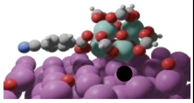	Zr-Bi (1)	2246.8	-1.04	-0.27	3.75	3.99	1.17
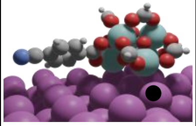	Zr-Bi (2)	2252.2	-1.06	-0.38	3.83	4.89	1.17

Section S2: Supporting Figures and relevant discussion.

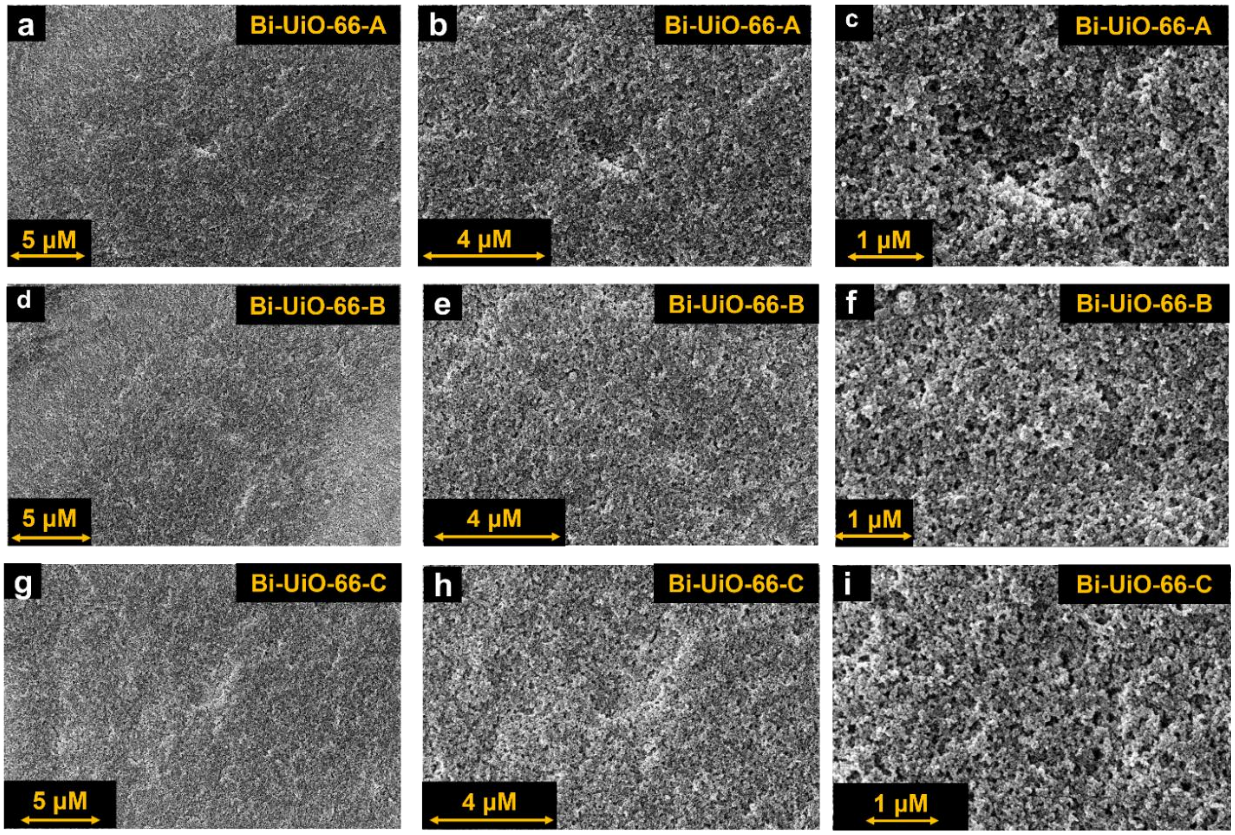

Figure S3. {(a), (b), and (c)}, {(d), (e), and (f)}, {(g), (h), and (i)}, represents top view SEM images of Bi-UiO-66-A, Bi-UiO-66-B and Bi-UiO-66-C, respectively.

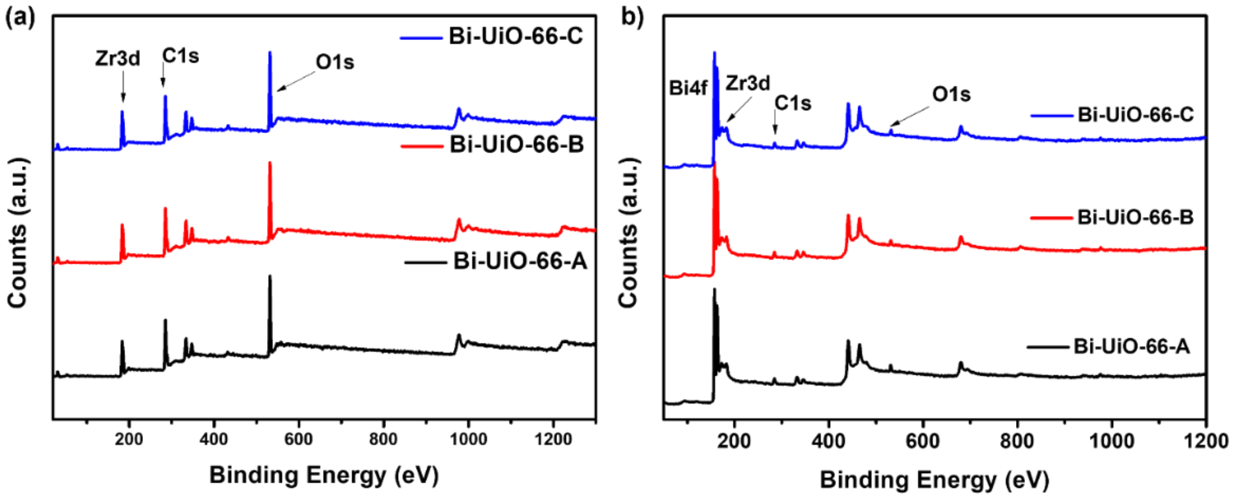

Figure S4. (a) XP surface survey scans without ion beam etching and (b) XP survey scans after ion beam etching for 900 s for Bi-UiO-66-A, Bi-UiO-66-B, and Bi-UiO-66-C, respectively.

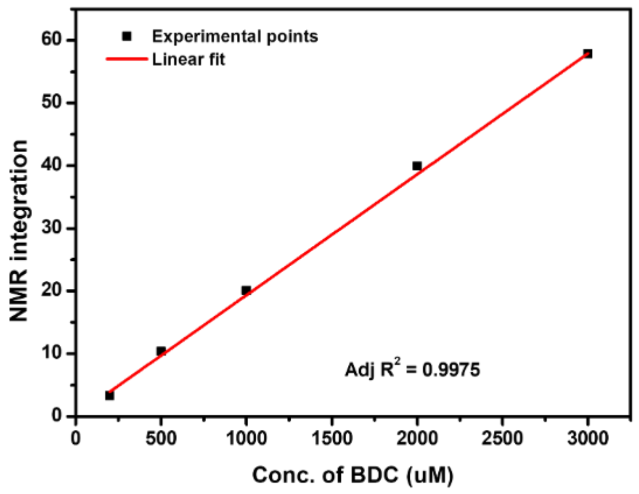

Figure S5. 1H-NMR calibration plot to determine the amount of benzene dicarboxylate (BDC) in UiO-66 and UiO-66-CN gel. The same calibration was used to determine the amount of −CN group in UiO-66-CN gel because under operational conditions the −CN group hydrolyses to −COOH group.

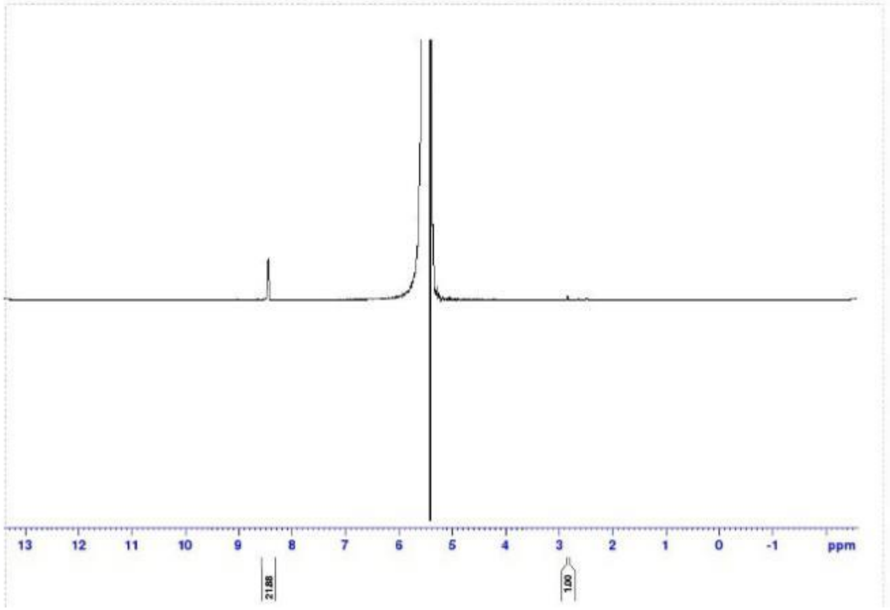

Figure S6. Representative digestive 1H-NMR of UiO-66 gel to determine the amount of BDC in UiO-66-gel.

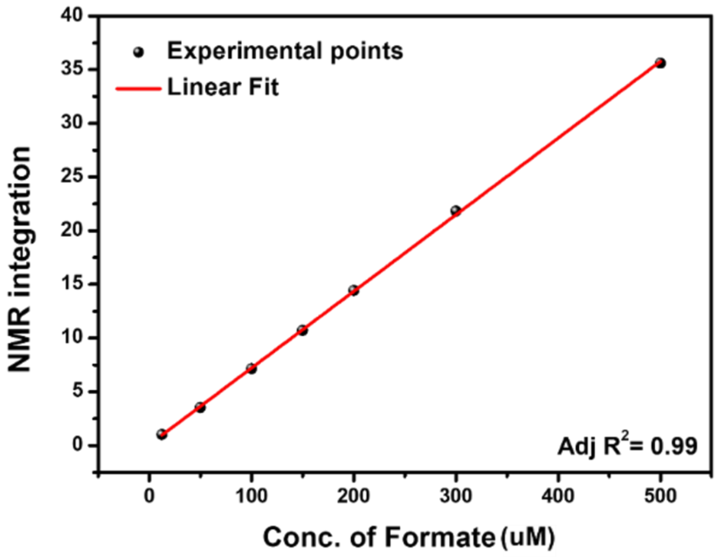

Figure S7. 1H-NMR calibration plot to determine the Faradic Efficiencies for HCOOH production (FE_{HCOOH}) for the different Bi-based catalysts. Calibration plot was prepared by recording NMR spectra of samples with a known concentration of HCOOH under identical conditions.

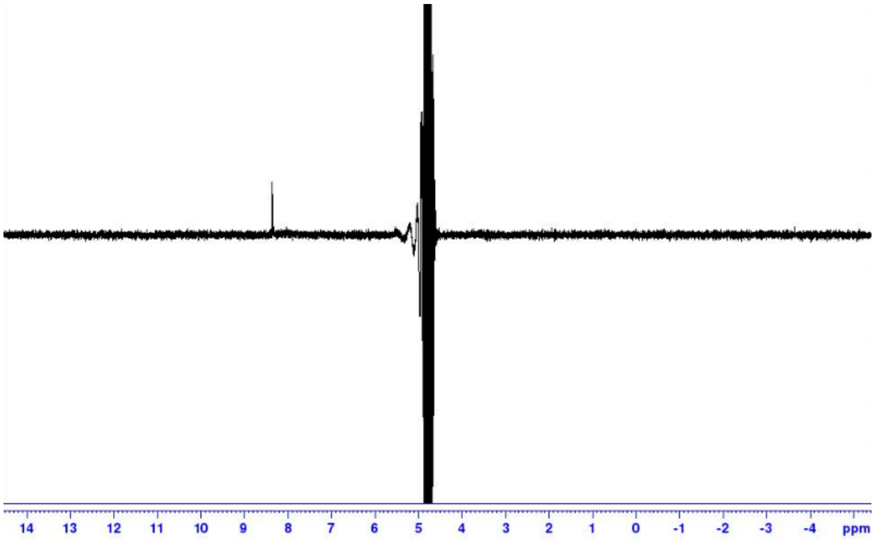

Figure S8. Representative 1H-NMR spectrum recorded for HCOOH quantification in case of Bi-UiO-66-B used as the catalyst for the CO_2 reduction reaction.

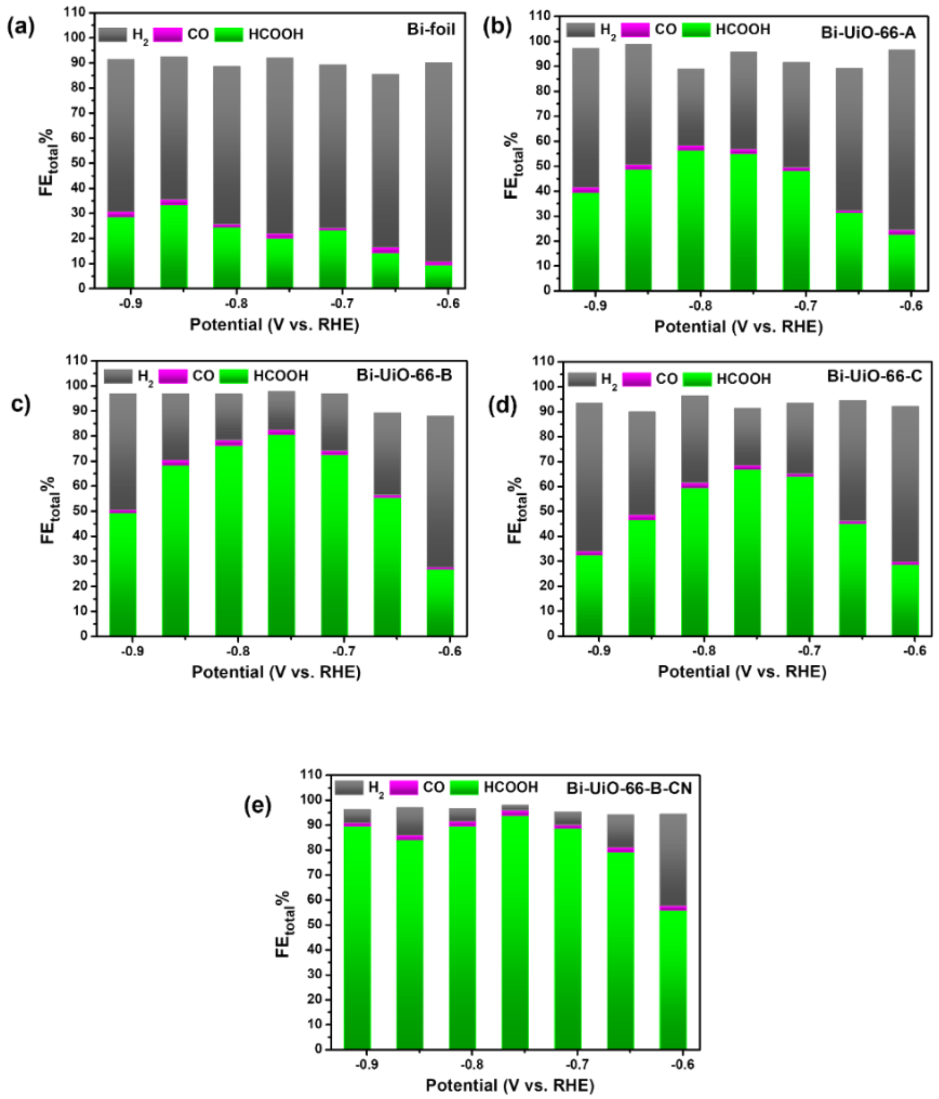

Figure S9. (a), (b), (c), (d), (e) show the total Faradic efficiencies (HCOOH + CO + H_2) for Bi-foil, Bi-UiO-66-A, Bi-UiO-66-B, Bi-UiO-66-C, Bi-UiO-66-B-CN, respectively. Electrolysis was performed in the potential range -0.6V to -0.9V (vs. RHE) in CO_2 saturated 0.1 M $NaHCO_3$.

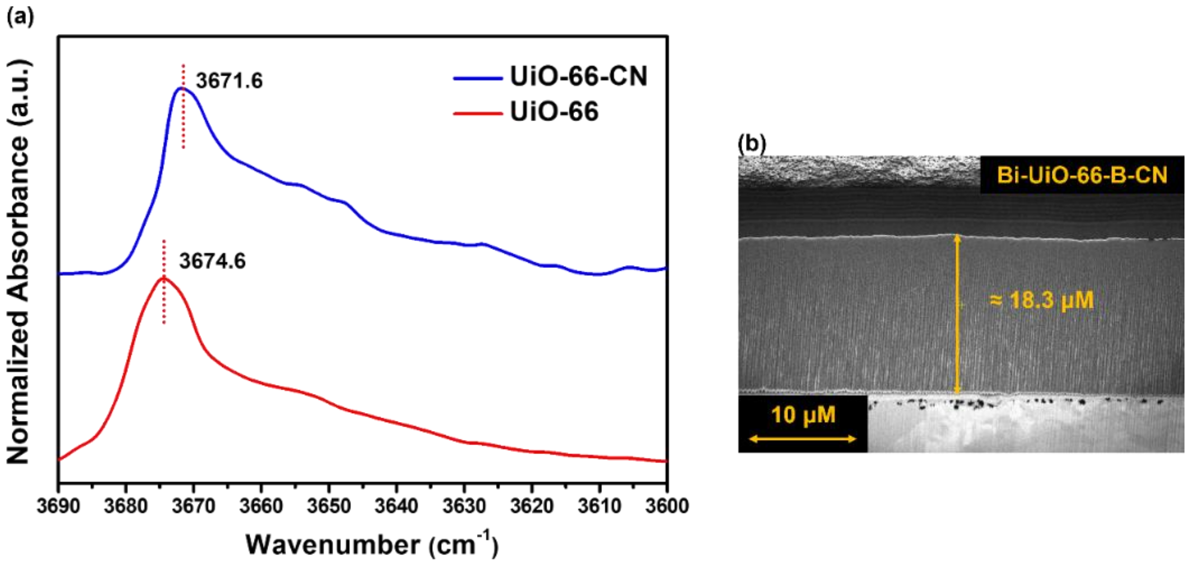

Figure S10. (a) DRIFTS spectra of UiO-66 and UiO-66-CN. In UiO-66-CN, the BA-CN occupies the terminal −OH groups of Zr_6-oxo nodes, causing a shift in the normalized spectra compared to UiO-66. (b) SEM-FIB image of Bi-UiO-66-B-CN showing a continuous UiO-66-CN membrane with a thickness of 18.3 μm on the Bi surface.

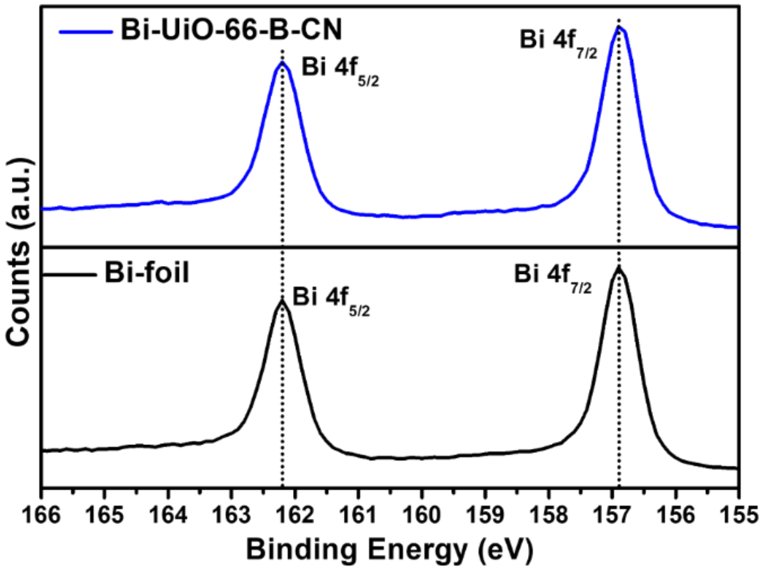

Figure S11. Bi 4f X-ray photoelectron spectra of Bi-foil and Bi-UiO-66-B-CN.

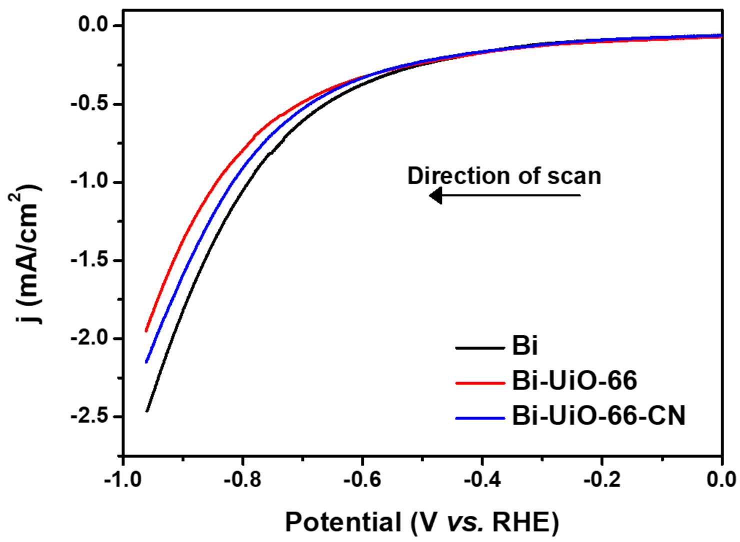

Figure S12. Linear sweep voltammetry of Bi, Bi-UiO-66 and Bi-UiO-66-CN under Ar in 0.1 M $NaHCO_3$. The scan rate was 100 mV/s.

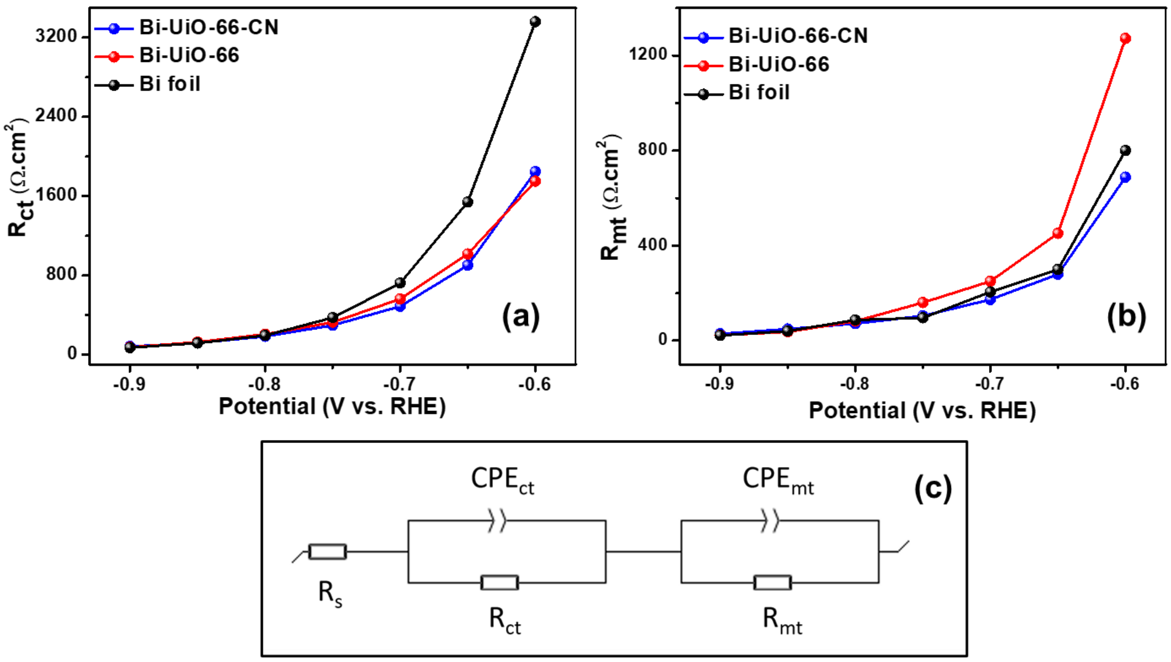

Figure S13. Electrochemical Impedance spectroscopy (EIS) analysis of Bi, Bi-UiO-66 and Bi-UiO-66-CN under different applied potentials, (a) Rct (charge transfer resistance), (b) Rmt (mass transport resistance), and (c) equivalence circuit used for fitting impedance data.

Impedance analysis of Bi, Bi-UiO-66 and Bi-UiO-66-CN.

EIS measurement of Bi, Bi-UiO-66 and Bi-UiO-66-CN was performed under different applied potentials. In all samples, we have detected the existence of 2 RC time-constants. A faster RC attributed to charge transfer at the Bi-electrolyte interface, as well as a slower RC, corresponding to diffusional mass-transport in solution .[14] We have analysed the data and plotted both Rct (charge transfer resistance) and Rmt (mass transport resistance) as a function of applied potential (see Figure S13). The charge transfer resistance (Rct) for Bi foil is larger than for Bi-UiO-66 and Bi-UiO-66-CN, thus signalling for acceleration of catalysis rate (e.g. higher catalytic currents) for the MOF-coated samples (Figure S13a). As for Rmt, we see that Bi-UiO-66 exhibits the highest mass transport resistance, while both Bi foil and Bi-UiO-66-CN have similar Rmt values. Meaning, compared to bare Bi foil, UiO-66 coated Bi shows higher resistance for diffusional mass transport in solution (in accordance with attenuation of diffusional mass transport through the UiO-66 membranes). Yet, UiO-66-CN coated Bi exhibits similar mass transport resistance as Bi foil, presumably due to the high local CO_2 concentration within the UiO-66-CN membrane, thus enabling larger reactant delivery toward the catalytic surface (Figure S13b). the existence of MOF-membrane coating on the Bi foil surface does not significantly limit diffusional mass transport of ions and catalytic-substrates toward the catalytically active sites.

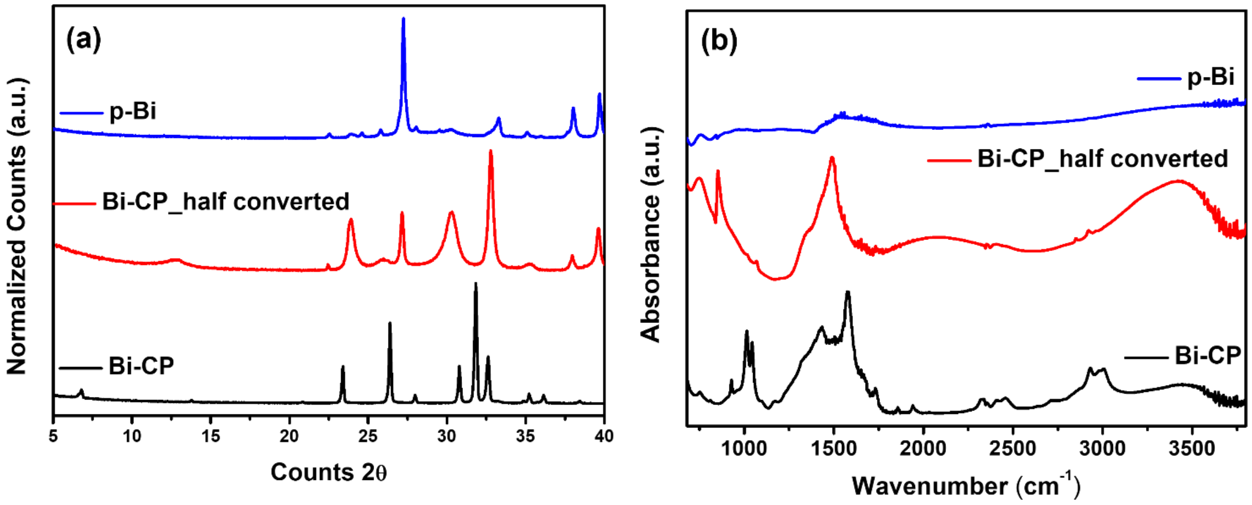

Figure S14. (a) PXRD patterns and (b) DRIFTS-IR spectra of Bi-CP, Bi-CP_half converted and p-Bi show the gradual chemical conversion from Bi-CP to p-Bi during chronoamperometric treatment at −0.9 V (vs RHE).

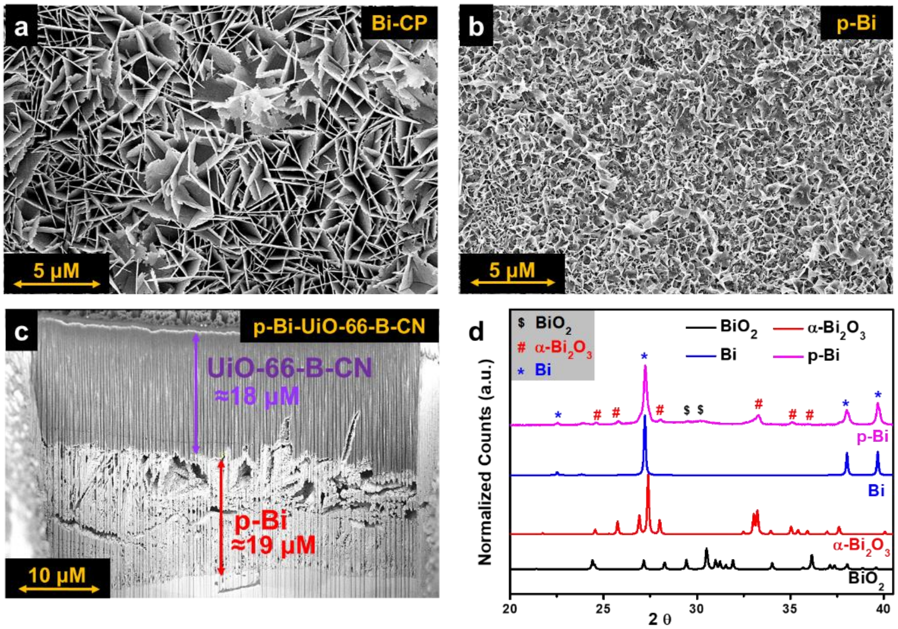

Figure S15. SEM images of (a) Bi-CP and (b) p-Bi. (c) cross-sectional SEM-FIB analysis of p-Bi-UiO-66-B-CN. (d) XRD analysis of p-Bi and comparing with simulated patterns of Bi, Bi_2O_3 and BiO_2.

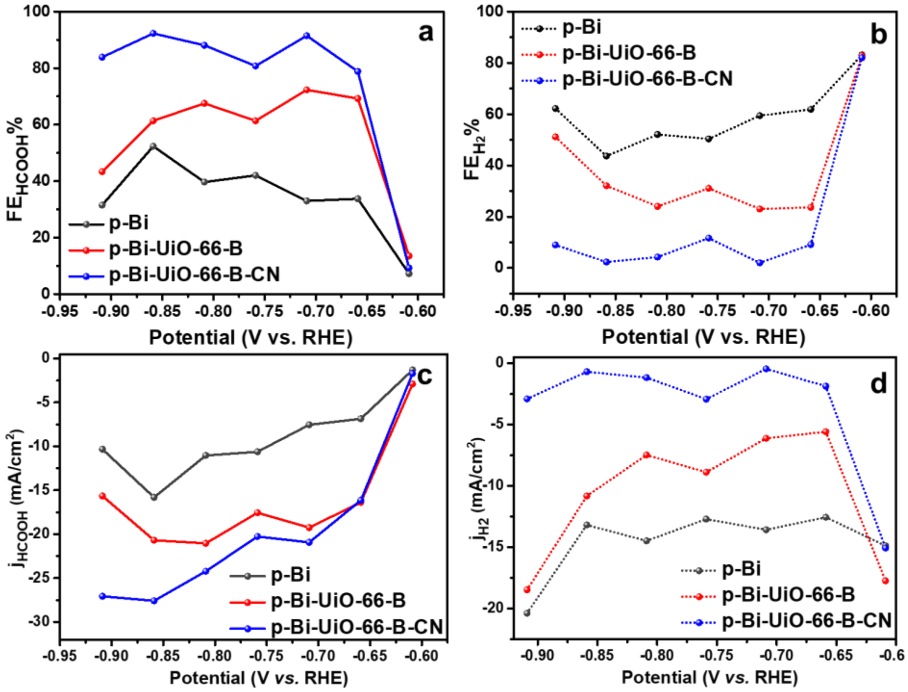

Figure S16. Variation in Faradaic efficiencies (FE) of p-Bi, p-Bi-UiO-66-B, and p-Bi-UiO-66-B-CN for (a) HCOOH and (b) H_2 at different applied potentials. Variation of partial catalytic currents of (c) HCOOH production (j_{HCOOH}) and (d) H_2 evolution reaction (j_{H2}) at different applied potentials.

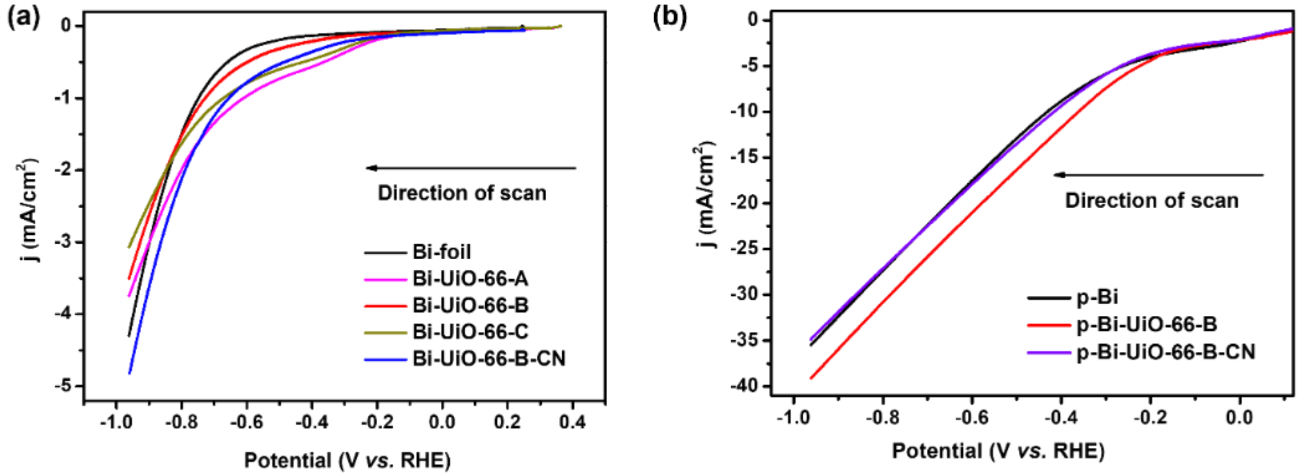

Figure S17. (a) Linear scan voltammograms (LSV) of Bi-foil, Bi-UiO-66-A-C, Bi-UiO-66-B-CN; (b) LSVs of p-Bi, p-Bi-UiO-66-B and p-Bi-UiO-66-B-CN. All measurements were done in CO_2 saturated 0.1 M $NaHCO_3$ using a two-compartment cell. The scan rate was 100 mV/s.

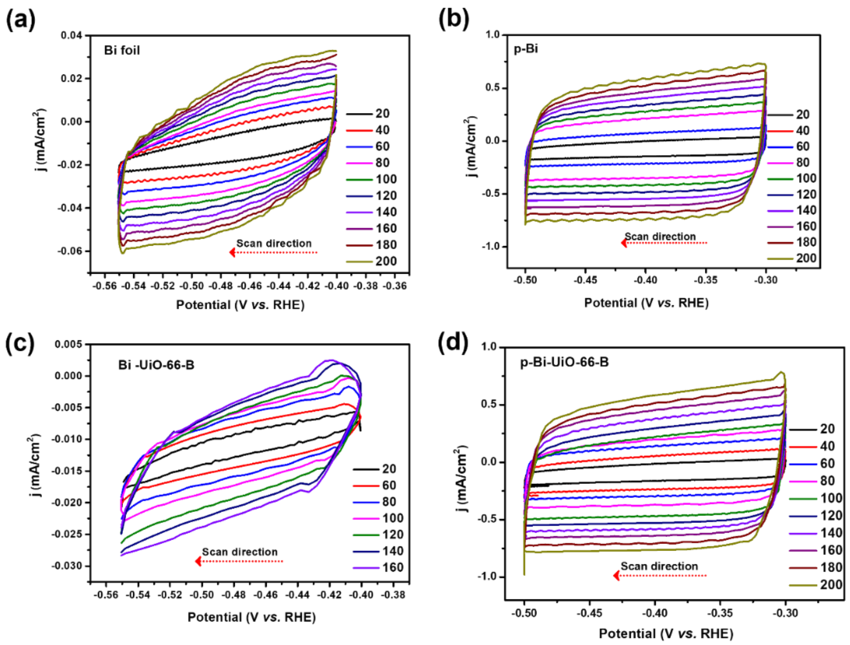

Figure S18. (a), (b), (c), (d), represents cyclic voltammograms recorded at the non-faradic region with different scan rates for Bi-foil, p-Bi, Bi-UiO-66 and p-Bi-UiO-66-B, respectively.

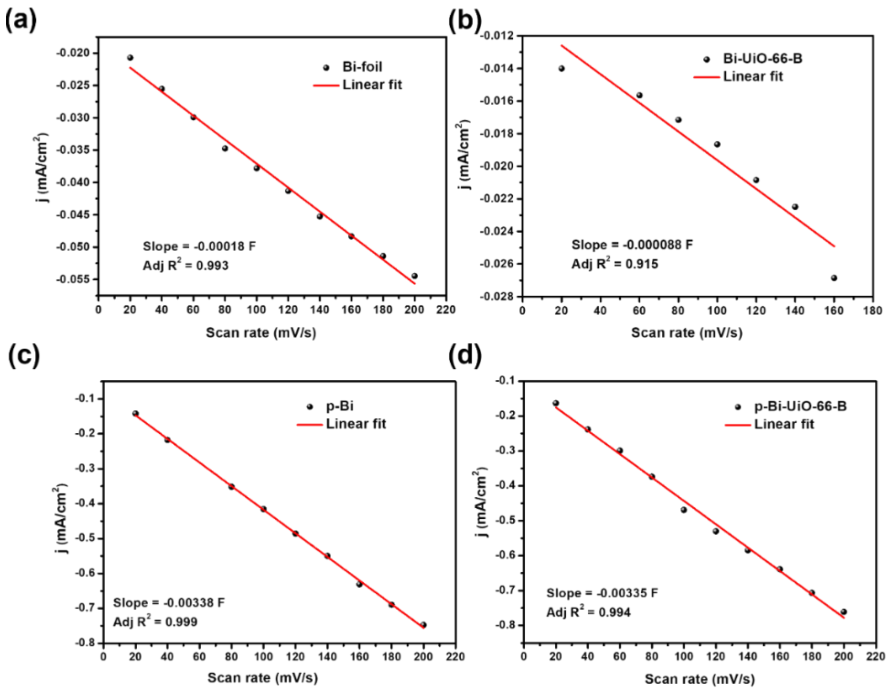

Figure S19. (a), (b), (c), (d), represents linear fit for the cathodic current @ −0.4 V (*vs.* RHE) for Bi-foil, Bi-UiO-66, p-Bi and p-Bi-UiO-66-B, respectively.

Electrochemical surface area calculation.

The electrochemically active surface area (EASA) of an electrode increases with an increase in the double-layer charge capacitance (C_{dl}). Thus, the relative order of EASA of the Bi-foil and the Bi-UiO-66-B can be understood from their respective C_{dl} values. Similarly, the relative order of EASA of the p-Bi and the p-Bi-UiO-66-B can be understood from their individual C_{dl} values. From the cyclic voltammograms (Figure S18) recorded at different scan rates in the non-faradic region using, the C_{dl} values can be realized. It involves the logical assumption that double-layer charging is the only process in this potential range. The slope of the plot between scan rate vs current density at this region gives the double-layer capacitance (C_{dl}) (Figure S19). The slope of the straight line for each case is provided below in a tabular form (Table S9).

Thus, the C_{dl} values corresponding to Bi-foil and Bi-UiO-66-B are similar, to be more specific, the C_{dl} for Bi-UiO-66-B was lower than that of Bi-foil. So, growing the UiO-66 membrane on Bi-foil did not increase the electrochemical surface area of Bi-foil. Similarly, the EASA for p-Bi and p-Bi-UiO-66-B would be very close to each other. Important to mention that the EASA corresponding to p-Bi and p-Bi-UiO-66-B is approximately an order of magnitude higher compared to Bi-foil and Bi-UiO-66-B.

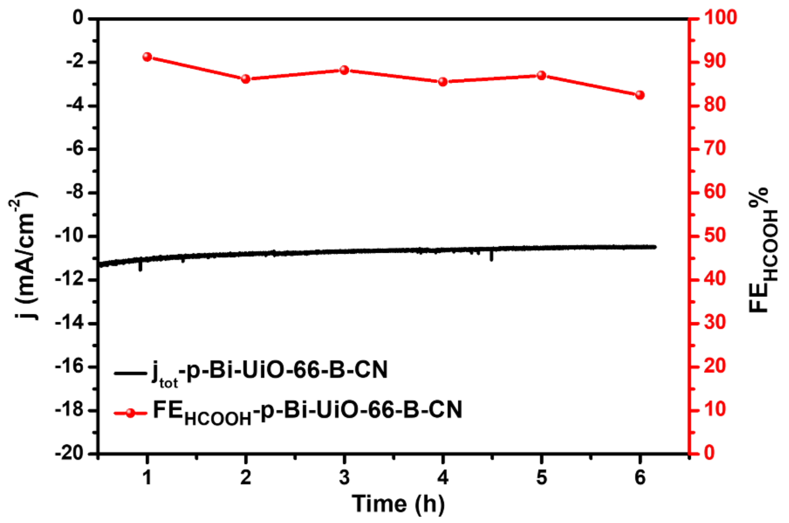

Figure S20. Variation of catalytic current density and HCOOH selectivity for 6 hours chronoamperometric measurement of p-Bi-UiO-66-B-CN in CO_2 saturated 0.1 M $NaHCO_3$ solution. To maintain the CO_2 saturation, the solution was purged with CO_2 continuously.

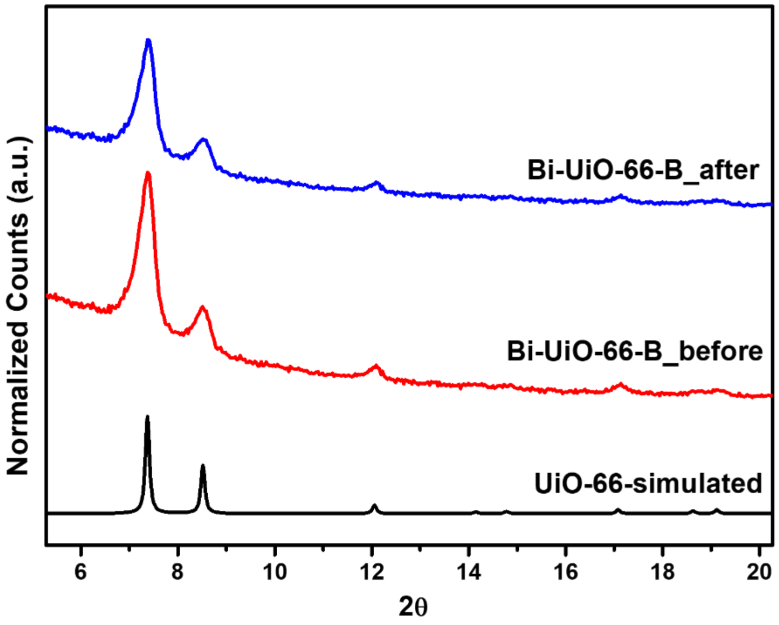

Figure S21. Comparing powdered X-ray diffraction pattern of p-Bi-UiO-66-B-CN before and after bulk electrolysis for 6 hours with the simulated XRD pattern of UiO-66.

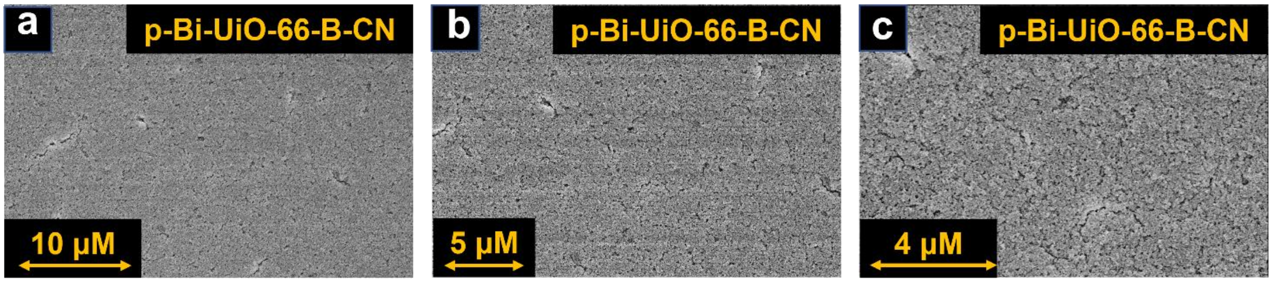

Figure S22. SEM (top view) images of p-Bi-UiO-66-B-CN. SEM images were recorded after bulk electrolysis for 6 hours using p-Bi-UiO-66-B-CN for electrochemical CO_2 reduction reaction.

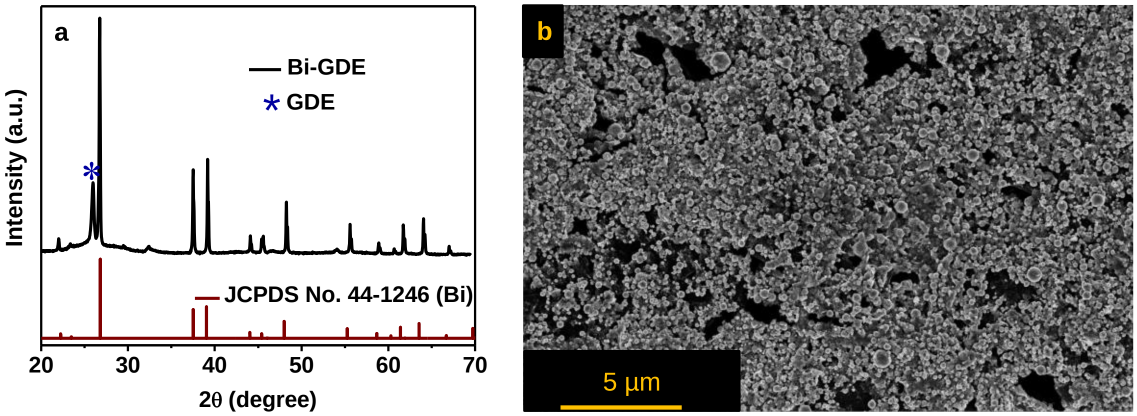

Figure S23. (a) PXRD pattern of Bi NPs coated GDE (Bi-GDE) compared with the reference spectrum Bi and (b) SEM image of Bi NPs on GDE.

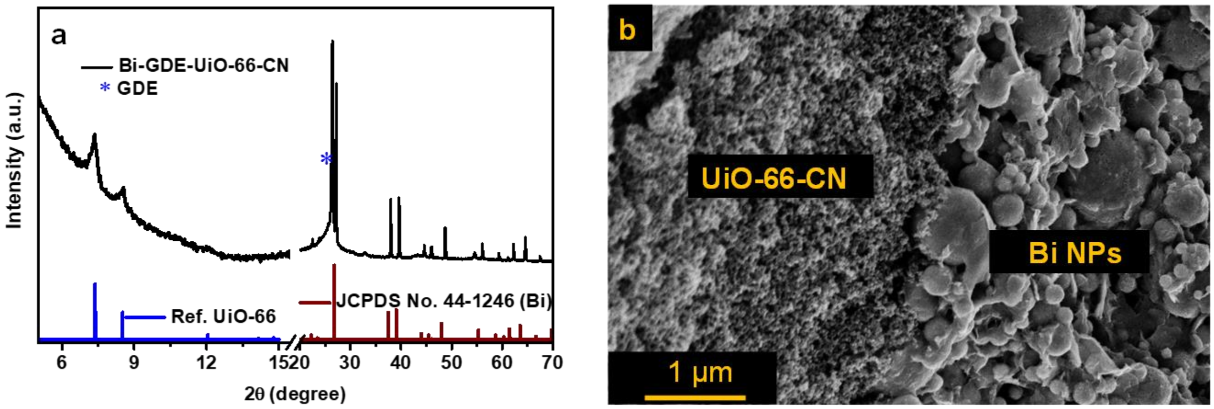

Figure S24. (a) PXRD pattern of Bi-GDE-UiO-66-CN compared with the reference spectra of Bi and UiO-66, separately and (b) SEM image of UiO-66-CN membrane over Bi NPs on GDE.

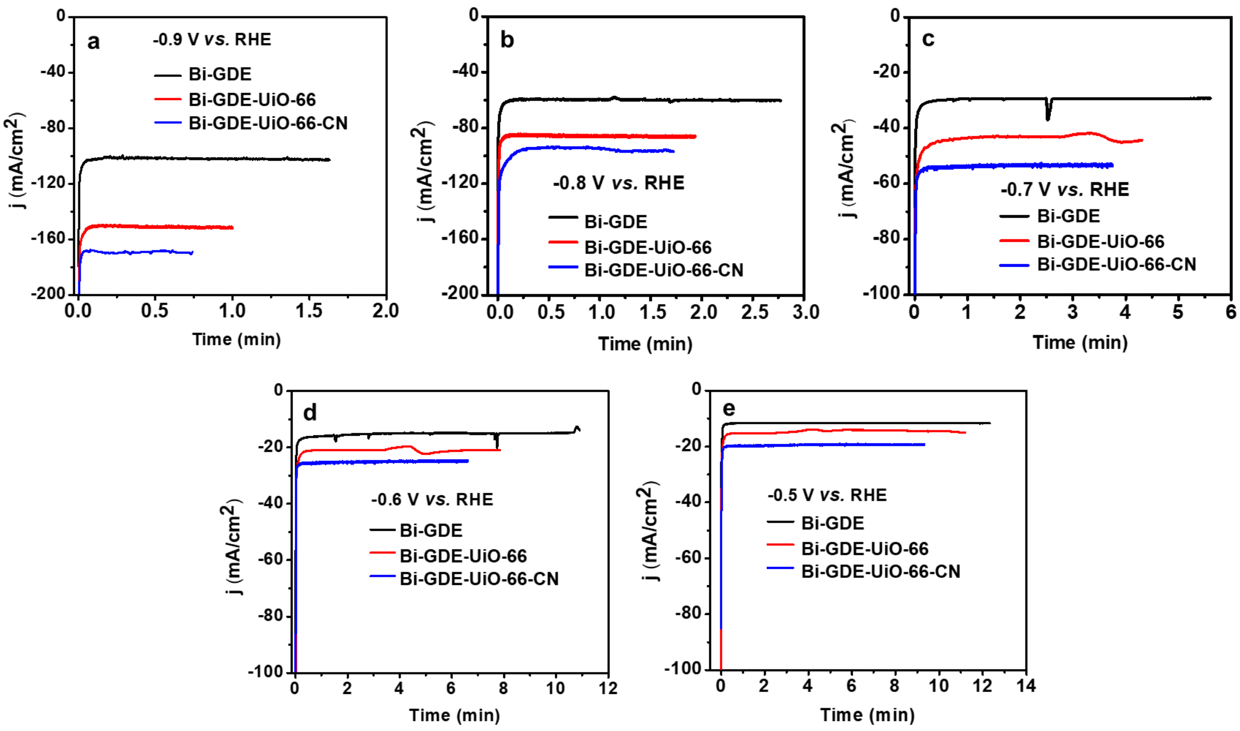

Figure S25. Chronoamperometric experiments were performed at a different applied potential of (a) -0.9 V vs RHE, (b) -0.8 V vs RHE, (c) -0.7V vs RHE, (d) -0.6 V vs RHE and (e) -0.5 V vs RHE.

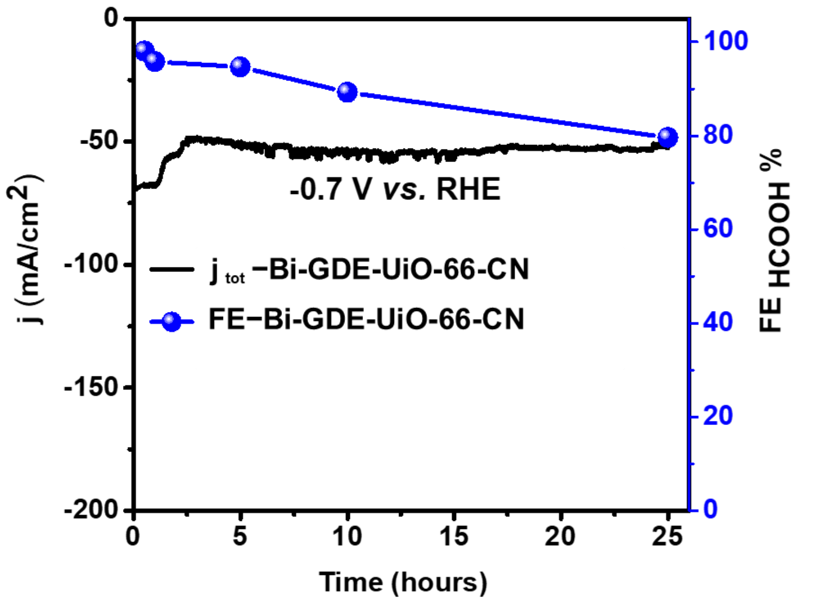

Figure S26. Variation of catalytic current density (black) and HCOOH selectivity (blue) for 25 hours. Chronoamperometric measurement of Bi-GDE-UiO-66-CN was performed by circulating CO_2 saturated 2.2 M $NaHCO_3$ electrolyte solution at a rate of 15 mL/min using pump through both catholyte and anolyte compartments separately, and a constant CO_2 flow was maintained throughout the measurement.

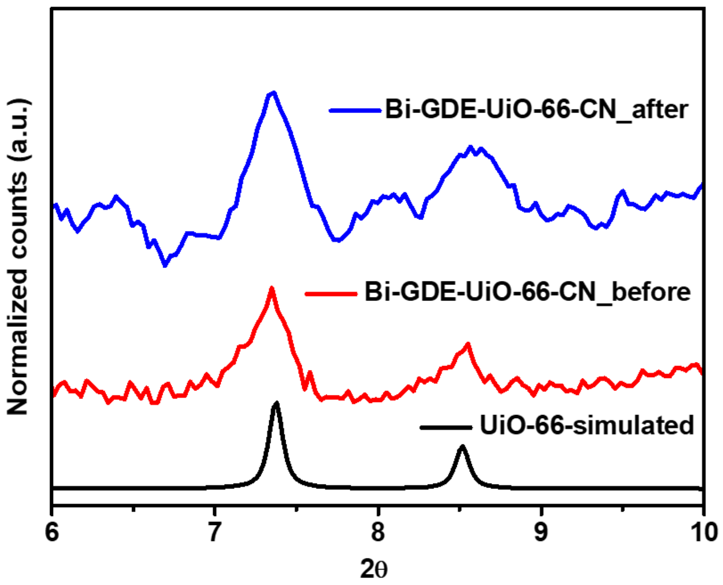

Figure S27. XRD pattern of Bi-GDE-UiO-66-CN UiO-66-CN before and after bulk electrolysis (25 h) experiment are compared with the simulated UiO-66 XRD pattern.

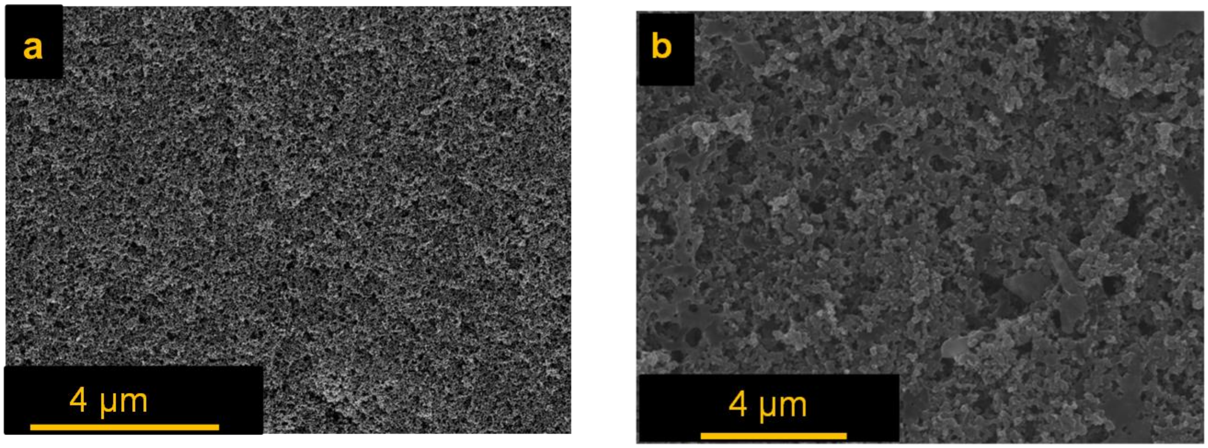

Figure S28. SEM images of Bi-GDE-UiO-66-CN UiO-66-CN before (a) and (b) after bulk electrolysis (25 h) experiment.

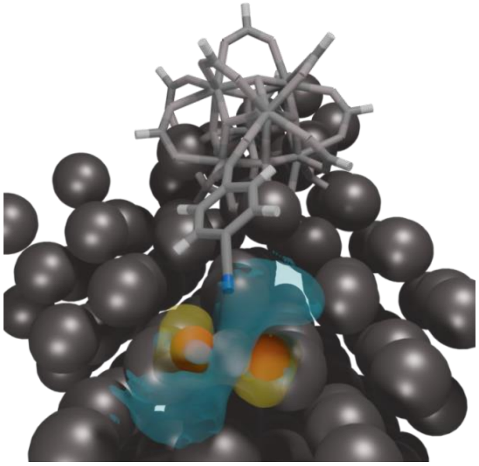

Figure S29: Charge density difference profile of CO_2 (isosurface level = 0.0024 e/Å^3. Yellow and blue indicate negative and positive charge, respectively) with Bi-UiO-66-CN indicates CO_2 activation due to its interaction with CN group leading to difference in charge distribution.

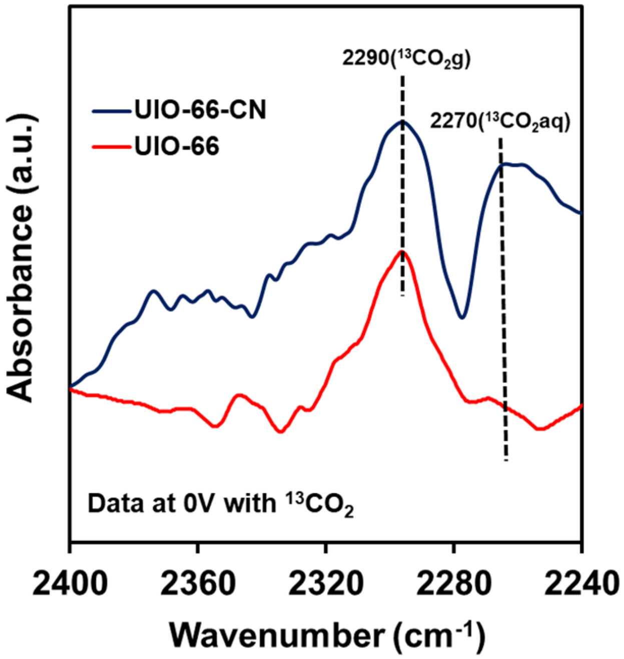

Figure S30. ATR-IRAAS spectra of UIO-66-B(red) and UIO-66-B-CN (blue) coated Bi electrode at 0V vs. RHE under operando condition in presence of $^{13}CO_2$-saturated 0.1 M $NaHCO_3$ aqueous solution in the CO_2 region.

ATR-IRRAS measurement of UiO-66-B and UiO-66-B 965-CN under 0V potential.

In order to confirm the CO_2 solubility peak, operando electrochemical study was performed using isotope labelled $^{13}CO_2$. Both UiO-66-B and UiO-66-B-CN membrane coated Bi electrodes were subjected to ATR-IRAAS analysis in 0.1 M $NAHCO_3$ aqueous solution after purging with $^{13}CO_2$ gas for 20 minutes at the

potential 0V vs. RHE, i.e. the potential where CO_2 reduction doesn't take place ensuring the solubility of CO_2 can be determined without perturbation. It has been found with UiO-66-B (Figure S30, red) a broad peak at 2290 cm^{-1} is generated, which corresponds to the gaseous $^{13}CO_2$ peak along with a very small peak at 2270 cm^{-1} corresponding to dissolved $^{13}CO_2$ in water. For UiO-66-B-CN, the peak at 2290 cm^{-1} is sharp while the peak at 2270 cm^{-1} is higher in intensity compared to that for UiO-66, indicating higher solvation of $^{13}CO_2$ in presence of ─CN group. The corresponding 70 cm^{-1} wavenumber $^{12/13}CO_2$ shift is in well accordance with the previous literature report[15] and therefore confirms the increase in CO_2 solubility in presence of ─CN without doubt.

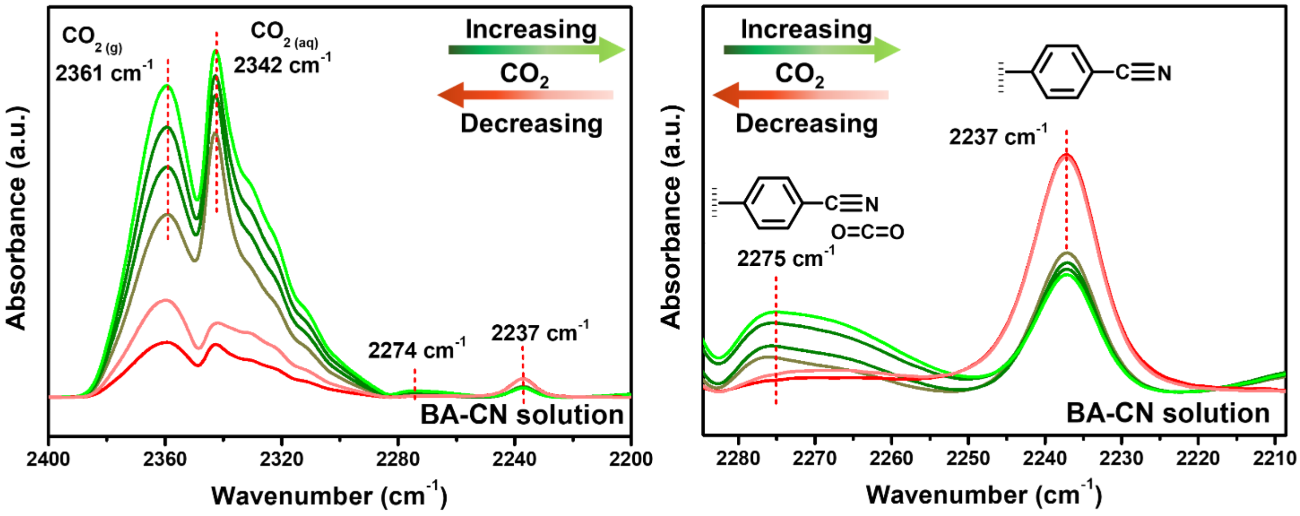

Figure S31. ATR-IRRAS spectrum of the aqueous solution of BA-CN (left) prepared in 0.1 M $NaHCO_3$. The effect of increasing and decreasing CO_2 concentration was studied by purging CO_2 and Ar in cycles in the aqueous solution. In the right, zoomed in ATR-IRRAS spectrum of UiO-66-CN depicting the changes in the peak intensity of BA-CN along with CO_2 concentration variation. The effect of increasing and decreasing CO_2 concentration was studied by purging CO_2 and Ar in cycles in the aqueous solution.

Use of quinone as redox probe to electrochemically determine CO_2 solubility for UiO-66-CN.

Usually, quinones are known to be one of the most well-studied examples as an organic redox couple. It is known that, in aqueous medium, quinones are electrochemically reduced to hydroquinones by consuming the protons, and the redox reaction involves $2H^+$, $2e^-$ process. To probe the CO_2 solubilization, the local concentration of CO_2 is estimated using the quinone redox couple by employing cyclic voltammetry (CV). CV data were collected for 1 mM 1,4 benzoquinone in an aqueous solution of 0.1 M $NaHCO_3$ under Ar and CO_2 atmospheres, respectively. Glassy carbon (GC) and thin layer UiO-66-CN coated GC (GC- UiO-66-CN) were used as working electrodes. Platinum flag and Ag/AgCl (saturated KCl) were used as counter,

and reference electrodes, respectively. Figure S32 corresponds to the CV data for 1 mM 1,4 benzoquinone in 0.1 M $NaHCO_3$ aqueous solution. Glassy carbon (GC) electrode and GC modified with UiO-66-CN membrane atop (named as GC-UiO-66-CN) were used as working electrodes for different measurements performed at scan rate of 50 mV/s under Ar or CO_2 atmosphere. CV results clearly suggest that the redox wave follows two electrons path in aqueous solution under both Ar and CO_2 atmosphere. In prior reports, the CO_2 binding constants were calculated for different CO_2 local concentration from the equation given below by extracting ΔE (difference in $E_{1/2}$ values under N_2 and CO_2 environment) from cyclic voltammetric experiments.[16]

$$E = E^o + \left(\frac{RT}{nF}\right) ln\left\{1 + [CO_2]K_Q\right\}$$

Where, R is universal gas constant, T is temperature (K), n is number of electrons involved during redox reaction, F is Faraday constant (C), and K_Q is binding constant.

Note: Number of electrons involved in the quinone redox reaction was 2, and the binding constant (K_Q) was assumed the same on both GC, and GC-UiO-66-CN catalysts as the same glassy carbon surface is the binding site here considering the fact that UiO-66-CN membrane is not active in nature. Thus, the modified equation was as follows.

$$\frac{\left[e^{\left(\frac{\Delta E}{12.59}\right)}\right]_{GC-UiO-66-CN}}{\left[e^{\left(\frac{\Delta E}{12.59}\right)}\right]_{GC}} = \frac{[CO_2]_{GC-UiO-66-CN}}{[CO_2]_{GC}}$$

Using the above equation, we have predicted the relative increase of CO_2 local concentration in the presence of UiO-66-CN from the ΔE parameter (difference in $E_{1/2}$ values in CO_2 environment compared to Ar environment) obtained from the CV data of GC, and GC-UiO66-CN, as shown in Table S10.

From the calculation, it is evident that the UiO-66-CN membrane layer could increase the CO_2 local concentration by ~ 32 times as compared to bare GC electrode.

Thus, we could prove the CO_2 solubilization effect of the UiO-66-CN membrane with an electrochemical technique, which is independent of the ATR-IRRAS measurements.

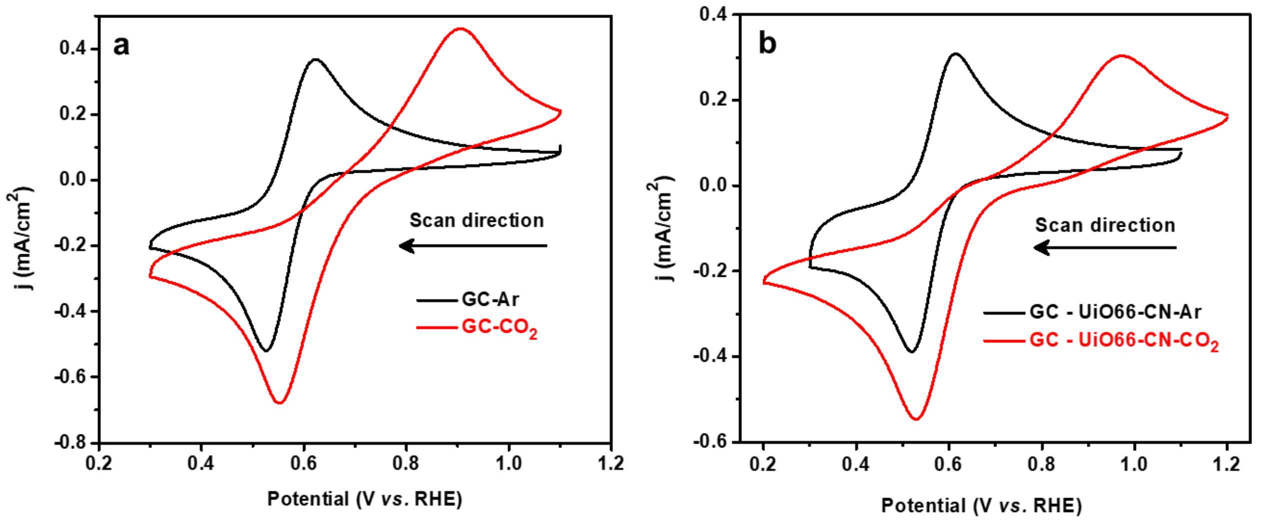

Figure S32. (a) and (b) cyclic voltammograms (CVs) of 1 mM 1,4 benzoquinone on GC, and GC-UiO-66-CN in 0.1 M $NaHCO_3$ aqueous electrolyte solution under Ar and CO_2 atmosphere.

Applicability of the electrochemical measurement using quinone as redox probe for quantification of CO_2.

Quinones are well-studied examples of organic redox couples in aqueous and non-aqueous electrolyte solutions.[17] It is well documented in the aqueous solution, quinones are electrochemically easily reduced to hydroquinones by consuming protons from the electrolyte solution ($2H^+$, $2e^-$) under Ar atmosphere, which produces well-defined symmetrical redox couple in cyclic voltammetry (CV). However, the redox properties of quinones were also investigated in aqueous solution in presence of saturated CO_2 atmosphere. Under such condition, in the electrochemical reduction process of quinones, CO_2 gets bound to its anionic form along with protons which increases ΔEp (peak to peak separation) along with peak width at half maxima and also shifts cathodic and anodic peak potentials individually in the CV data of quinone compared to that in Ar atmosphere.[18] From the obtained ΔEp values in the CV data of quinone in saturated CO_2 vs. Ar, the concentration of bound/adsorbed CO_2 on the catalyst surface can be determined according to the equation given in ref. 47 (main manuscript). This concept has also been further used for carbon capture and release experiments using an electrochemical approach.[19] Similarly, in our case for both electrodes (with and without UiO-66-CN), under CO_2 atmosphere, ΔEp increases in CO_2 atmosphere compared to that in Ar atmosphere for quinine redox couple, albeit to the fact that the increase in ΔEp is larger by 0.043V for UiO-66-CN MOF (see Figure S32). This clearly suggests, that the exposed -CN functional group of UiO-66-CN has a role in the enhancement of CO_2 binding/adsorption at the electrode.

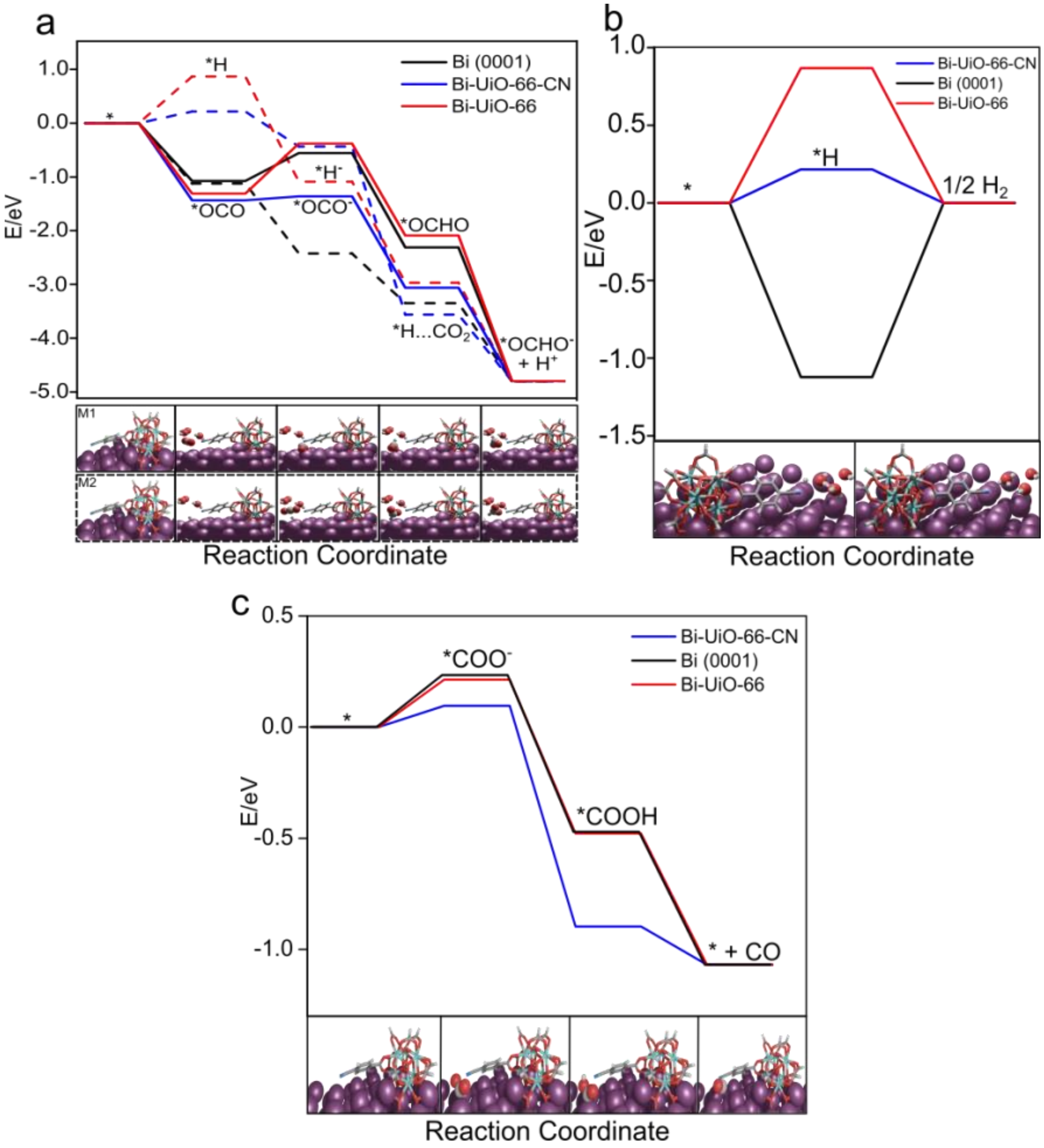

Figure S33. (a) M1 and M2 reaction profiles for all interfaces including explicit solvation. The solid lines represent HCOOH production via CO2 adsorption and activation followed by a PCET step (M1) and dashed lines represent Heyrovsky mechanism (M2) with initial hydride formation on the surface. The solvation correction limits M2 for Bi-UiO-66 and Bi-UiO-66-CN. (b): HER reaction profile for all three interfaces. (c): CO production reaction profile for all three interfaces. The CO2 activation (*COO-) requires an energy penalty in all three interfaces making the process unfavorable.

Use of −CN stretching peak as a probe to support the existence of interaction between −CN and *OCHO intermediate and between −CN and CO_2.

We have followed the −CN stretching region during the operando ATR-IRRAS analysis using the same method described in the method section of the main text, within the potential window of -0.6 V to -0.9 V (vs RHE) with 50 mV intervals between each measurement. Indeed, the peak position for −CN has been found to be a suitable probe for the interaction of nitrile with the intermediate. Figure 8d (main text), represents the IR data of Bi-UiO-66-CN MOF-membrane system, where a gradual increase of a set of three peaks at 2237 cm^{-1}, 2265 cm^{-1}, and 2295 cm^{-1}, respectively are observed in the difference spectra (auto subtracted from the background spectrum obtained at 0 V) as we keep on applying the cathodic potential. The first peak at 2237 cm^{-1}, represents the −CN stretching frequency of free nitrile, i.e., not bound to CO_2 or *OCHO intermediate, which is in accordance with the previous literature report,[20] while the peak at 2265 cm^{-1} indicates the presence of CO_2-interacted nitriles. These peak positions also match well with the peak of −CN and the peak indicative of interaction between −CN and CO_2 as obtained from the ATR-IRRAS measurements for CO_2 solvation without applying potential (Figure 6, main text). Further closer scrutiny of Figure 8d (main text) shows another prominent increase of a broad peak at 2295 cm^{-1}, with increasing cathodic potential. We assume the interaction between *OCHO intermediate and the −CN group is responsible for the peak. All the mentioned peak positions were further confirmed by DFT.

The variation of the peaks with the increasing cathodic potential provided us important information about the CO_2 reduction process. During the catalysis, the catalyst-bound *OCHO intermediate may accumulate over the electrode surface. With increasing cathodic potential, the peak area corresponding to the interaction between −CN and *OCHO (2295 cm^{-1}) increases gradually, which signifies enhanced CO_2 reduction reaction to cause rapid generation of the intermediate *OCHO on the catalytic surface. Rapid accumulation of *OCHO intermediate should accompany with fast depletion of CO_2. If that was the case, the peak positioned at 2265 cm^{-1} (interaction between CO_2 and −CN) should decrease. Contrary to this, as the potential is gradually increased to higher cathodic values, the peak does not decrease, in fact, it grows slightly. To understand the observation, we need to keep in mind that in the ATR-IRRAS measurement, we see the response not only from the Bi−MOF interface (the catalytic surface) but also a few immediate layers of the MOF membrane (non-catalytically active CO_2 solvation layer). We can assume that the CO_2 solvation layer of the MOF will always attempt to maintain a high CO_2 concentration near the catalyst surface and replenish the consumed CO_2. As the consumption of CO_2 increases with increased cathodic potential, CO_2 concentration in the catalyst adjacent few layers of the MOF also increase, causing the peak at 2265 cm^{-1} to grow. A similar situation is also observed for the free −CN peak. The peak due to the free −CN groups (at 2237 cm^{-1}) should normally increase significantly with increasing cathodic potential, because of the cumulative effect of rapid consumption of CO_2 and release of *OCHO intermediates into HCOOH. However, the increase in the peak area for the peak at 2237 cm^{-1} is less pronounced due to the MOF layers. As we record the response for −CN groups present, in the MOF-Bi interface along with a few layers of the MOF in the ATR-IRRAS measurement, we see here an

averaged response of the (a) increasing amount of free unbound −CN groups at the Bi−MOF interface and (b) decreasing amount of unbound −CN groups at the adjacent layers of the MOF due to increased CO_2 concentration in these layers.

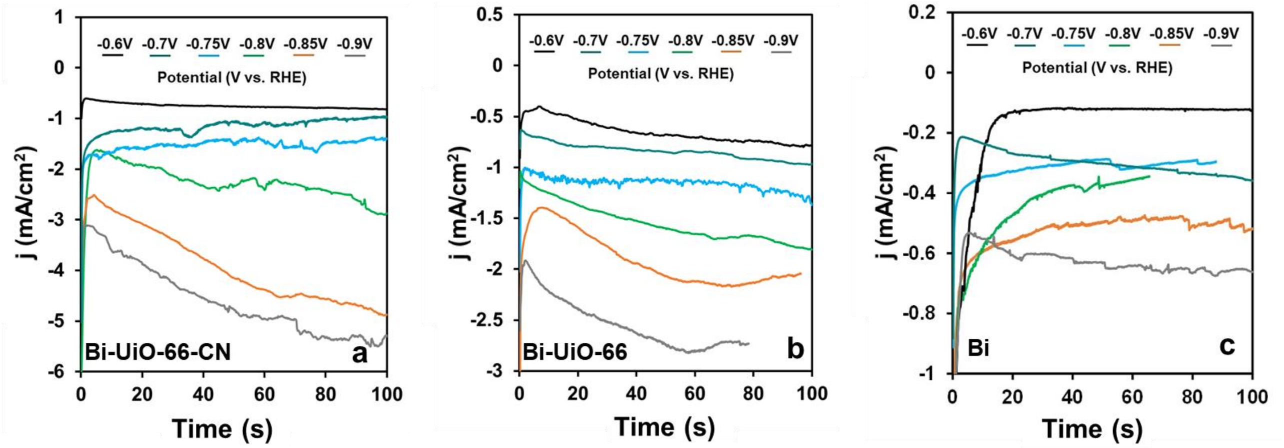

Figure S34. Chronoamperometric measurements for (a) Bi-UiO-66-CN, (b) Bi-UiO-66 and (c) Bi during ATR-IRRAS measurements. Each ATR-IRRAS spectrum was recorded after 60 seconds of time, so that the steady-state is attained.

Necessary outcome about the intermediate *OCHO stabilization obtained from the isotope labelling experiments.

To characterize the intermediate peaks from IR spectroscopy, we have performed operando spectroelectrochemical studies (Figure S35) with isotope labelled $^{13}CO_2$ and the corresponding DFT simulations. In the $^{12}CO_2$ saturated aqueous 0.1 M $NaHCO_3$ solution a gradual rise of a band at 1380 cm^{-1} is observed for Bi-UiO-66-CN by ATR-IRRAS measurement as we keep on applying more negative cathodic potential (Figure S35 g). Interestingly, for $^{13}CO_2$ saturated 0.1 M aqueous $NaHCO_3$ solution, rise of a band at 1335 cm^{-1} is observed for Bi-UiO-66-CN under increasing cathodic potential (Figure S35 h). The band at 1380 cm^{-1} for $^{12}CO_2$ and its 45 cm^{-1} shift (Figure S35 i) for $^{13}CO_2$ isotope labelled measurement matches well with that of the ν(C−O) of *OCHO species bound to Bi. As per DFT calculations, a similar shift of 31.4 cm^{-1} was observed between $^{13}CO_2$ and $^{12}CO_2$ for Bi-UiO-66-CN (Table S6). IR stretching frequency is inversely proportional to the reduced mass of the analyte, which causes the downshift of the specific vibration seen here. Similarly, for spectroelectrochemical measurements using isotope labelled $^{13}CO_2$, in case of Bi, and Bi-UiO-66, a shift in ν(C−O) band is observed from 1410 cm^{-1} to 1370 cm^{-1} (40 cm^{-1} shift) and 1400 cm^{-1} to 1354 cm^{-1} (46 cm^{-1} shift), respectively (Figure S35, a, b, c, d, e and f) due to the mass variation explained earlier. Both

these shifts are in well accordance with the DFT data where a shift of 39.0 cm^{-1} and 39.8 cm^{-1} for Bi (0001), and Bi-UiO-66, respectively was observed (Table S6). This $^{13}CO_2$-sensitive vibration (for all the three catalytic systems) provide direct evidence for the C−O bond of the bound *OCHO intermediate species, which in turn proves that the relative shift we are seeing in this region, i.e., 1410 cm^{-1} for Bi to 1400 cm^{-1} for Bi-UiO-66 and further to 1380 cm^{-1} for Bi-UiO-66-CN, is mainly due to different extent of stabilization of the intermediate caused by the MOF membrane.

It is to be noted that, along with the shifted band at 1335 cm^{-1}, the band at 1380 cm^{-1} was also present for the $^{13}CO_2$ isotope labelled spectroelectrochemical measurements for UiO-66-CN (Figure S35 i). The dissolved CO_2 can attain an equilibrium of the type: $HCO_3^- \leftrightarrow CO_2 + OH^-$ in 0.1 M $NaHCO_3$ solution.[21] Because of the dynamic equilibrium between the non-isotope labelled $NaHCO_3$ and isotope labelled $^{13}CO_2$, a mixture of $*O^{12}CHO$ and $*O^{13}CHO$ intermediate species are formed during the electrocatalysis, which leads to the formation of two bands in the ATR-IRRAS measurements. However, such a scenario was not observed for the isotope labelled spectroelectrochemical measurements involving only Bi and Bi-UiO-66-B. It can also be an indirect proof of probable increased residence time of the isotope labelled $^{13}CO_2$ near the electrode surface, caused by the higher local concentration of $^{13}CO_2$ due to the CO_2 solubilizing effect of UiO-66-CN MOF membrane. The intermediate peak with its experimental and theoretical shifts (calculated by DFT) is also tabulated in Table S11.

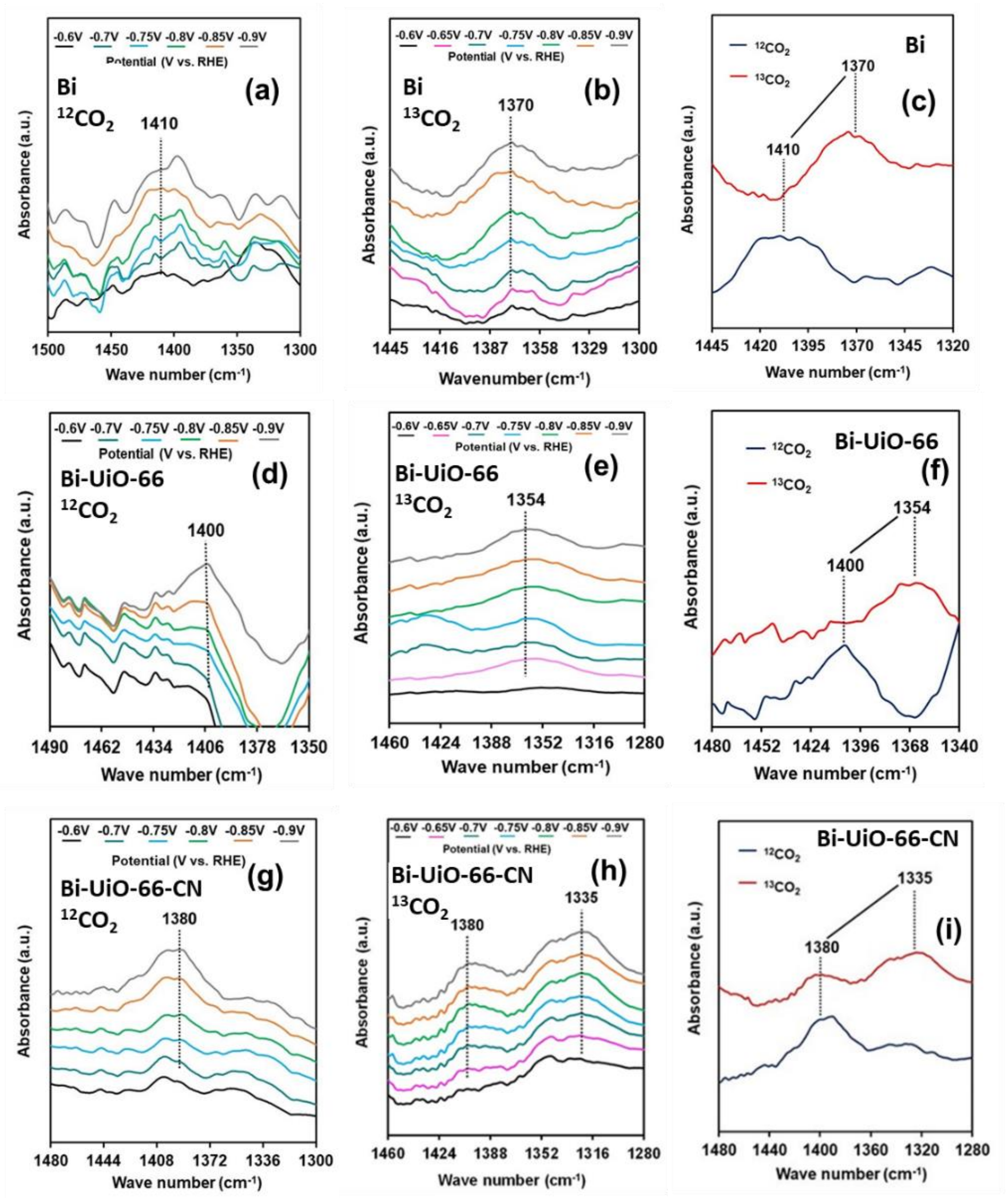

Figure S35. Operando electrochemical ATR-IRRAS spectra zoomed in to highlight the *OCHO intermediate region of Bi (a-c), Bi-UiO-66 (d-f), and Bi-UiO-66-CN (g-i) under electrochemical CO_2 reduction conditions with $^{12}CO_2$ (a, d, g) and $^{13}CO_2$ (b, e,h). (c, f, and i) represent the overlay of -0.9 V (vs. RHE) spectra of both $^{12}CO_2$ and $^{13}CO_2$ for Bi, Bi-UiO-66 and Bi-UiO-66-CN, respectively.

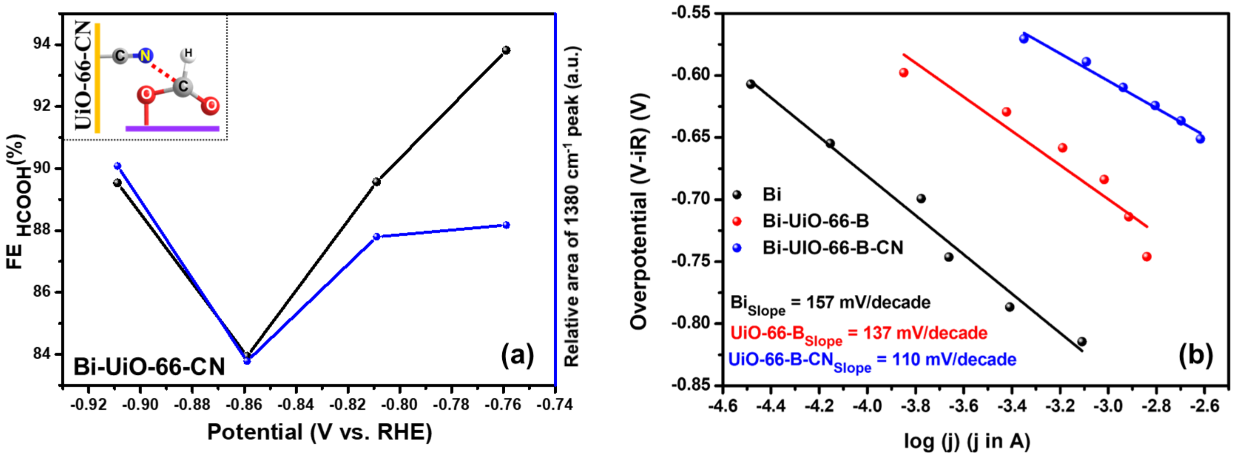

Figure S36. (a) For UiO-66-B-CN, the peak area at 1380 cm^{-1} and FE_{HCOOH} plotted against applied potential from −0.75 V to −0.9 V (vs RHE). The ATR-IRRAS spectrum recorded at 0 V was considered as the background and auto-subtracted from all the measurements. **(b)** Tafel plots for Bi, Bi-UiO-66-B, and Bi-UiO-66-B-CN.

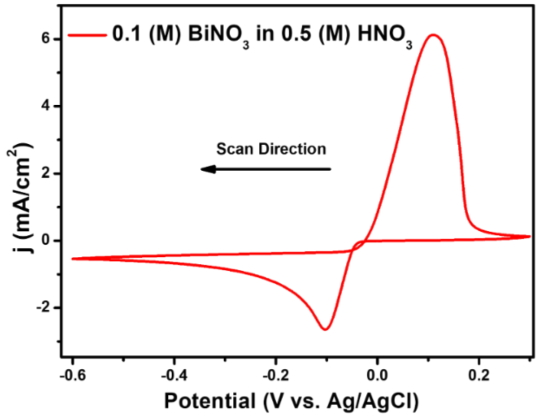

Figure S37. Cyclic voltammogram recorded for 0.1 (M) $BiNO_3$ solution prepared in aqueous 0.5 (M) HNO_3. Glassy carbon electrode, Ag/AgCl, Pt-flag electrode were used as the working, the reference and the counter electrodes, respectively. The scan rate was 100 mV/s.

Table S8. Comparison table for previous reports of high-performance CO_2 to HCOOH conversion using bicarbonate ($KHCO_3$) buffer with a three-electrode set-up in a GDE flow-cell.

Serial No.	Type of GDE electrode	Maximum catalytic current (mA/cm^2)	j_{HCOOH} (mA/cm^2)	Faradaic Efficiency for HCOOH (%)	Operational potential (V *vs.* RHE)	Reference
1	Bi-Sn Alloy	250		92	-1.15	22
2	$ZnIn_2S_4$		298	99	-1.18	23
3	SCuSn	250	241	96.4	-2.22	24
4	CuBi-HFGDE	141		85	-1	25
5	Sn-Cu	216		88.1	-1.1	26
6	Bi-In	120		80	NA	27
7	Cu-Sn	66.1		89	-1.11	28
8	Bi nanosheet	430		87	-1.51	29
9	**Bi-GDE-UiO-66-CN**	**188.5**	**166.6**	**88.4**	**-0.9**	**Our work**

Table S9. Slope corresponding to C_{dl} of Bi-foil, p-Bi, Bi-UiO-66-B and p-Bi-UiO-66-B.

**Sample**	**Slope of linear fit (Fcm^{-2})**
**Bi-foil**	1.8×10^{-4}
**Bi-UiO-66-B**	0.88×10^{-4}
**p-Bi**	33.8×10^{-4}

**p-Bi-UiO-66-B**	33.5 × 10^{-4}

Table S10. ΔE parameters obtained on GC, GC-UiO66-CN catalysts from CV under the Ar and CO_2 atmosphere.

Catalyst	$E_{1/2}$ (mV) in Ar	$E_{1/2}$ (mV) in CO_2	ΔE (mV)
**GC**	-25.6	128	153.6
**GC-UiO-66-CN**	-46.6	150	196.6

Table S11. Intermediate peaks for different catalysts systems

Catalyst systems	ν-(^{12}CO) of *OCHO (cm^{-1})	ν-(^{13}CO) of *OCHO (cm^{-1})	Isotope shift (Exp.) (cm^{-1})	Isotope shift (from DFT calculation) (cm^{-1})
**Bi-UiO-66-CN**	1380	1335	45	31.4
**Bi-UiO-66**	1400	1354	46	39.8
**Bi-only**	1410	1370	40	39.0

References

1. Gong, Q. *et al.* Structural defects on converted bismuth oxide nanotubes enable highly active electrocatalysis of carbon dioxide reduction. *Nat. Commun.* **10**, 2807 (2019).

2. Feng, X. *et al.* Bi_2O_3/BiO_2 Nanoheterojunction for highly efficient electrocatalytic CO_2 reduction to formate. *Nano Lett.* **22**, 1656-1664 (2022).

3. Zheng, W., Li, Y., Tsang, C.-S., So, P.-K. & Yoon Suk Lee, L. Stabilizer-free bismuth nanoparticles for selective polyol electrooxidation. *iScience* **24**(2021).

4. Faguy, P. W. & Marinkovic, N. S. Sensitivity and reproducibility in infrared spectroscopic measurements at single-crystal electrode surfaces. *Anal. Chem.* **67**, 2791–2799 (1995).

5. Faguy, P. W., Marinković, N. S. & Adžić, R. R. Infrared spectroscopic analysis of anions adsorbed from bisulfate-containing solutions on Pt(111) electrodes, *J. Electroanal. Chem.*, **407**, 209-218 (1996).

6. Li, M. *et al.*, Ethanol oxidation on the ternary Pt–Rh–SnO_2/C electrocatalysts with varied Pt:Rh:Sn ratios, *Electrochim. Acta,* **55**, 4331-4338 (2010).

7. Griffiths, P. R. & Haseth, J. A. Fourier transform infrared spectrometry. John Wiley & Sons, Inc. (2007).

8. Normand, E., Duxbury, G., & Langford, N. Characterisation of the spectral behaviour of pulsed quantum cascade lasers using a high-resolution Fourier transform infrared spectrometer. *Opt. Commun.,* **197**, 115-120, (2001).

9. Oklejas, V., Sjostrom, C., & Harris, J. M. SERS detection of the vibrational stark effect from nitrile-terminated SAMs to probe electric fields in the diffuse double-layer. *J. Am. Chem. Soc.* **124**, 2408–2409 (2002).

10. Delley, M. F., Nichols, E. M., & Mayer, J. M. Interfacial acid–base equilibria and electric fields concurrently probed by in situ surface-enhanced infrared spectroscopy. *J. Am. Chem. Soc.* **143**, 10778–10792 (2021).

11. Chang, X., *et al.* Determining intrinsic stark tuning rates of adsorbed CO on copper surfaces. *Catal. Sci. Technol.*, **11**, 6825-6831 (2021).

12. Kortlever, R., Shen, J., Schouten, K.J.P., Calle-Vallejo, F. & Koper, M.T.M. Catalysts and reaction pathways for the electrochemical reduction of carbon dioxide. *J. Phys. Chem. Lett.* **6**, 4073-4082 (2015).

13. Birdja, Y.Y. et al. Advances and challenges in understanding the electrocatalytic conversion of carbon dioxide to fuels. *Nat. Energy* **4**, 732-745 (2019).

14. Braz, B.A., Moreira, C.S., Oliveira, V.B. & Pinto, A.M.F.R. Electrochemical impedance spectroscopy as a diagnostic tool for passive direct methanol fuel cells. *Energy Rep.* 8, 7964–7975 (2022).

15. Schadle, T., Pejcicb, B., & Mizaikof, B. Monitoring dissolved carbon dioxide and methane in brine environments at high pressure using IR-ATR spectroscopy. *Anal. Methods*, *8,* 756-762 (2016).

16. Sampson, M. D., *et al.* Manganese catalysts with bulky bipyridine ligands for the electrocatalytic reduction of carbon dioxide: eliminating dimerization and altering catalysis. *J. Am. Chem. Soc.* 136, 5460–

5471 (2014).

17. Quan, M., Sanchez, D., Wasylkiw, M.F., & Smith, D.K. Voltammetry of Quinones in Unbuffered Aqueous Solution: Reassessing the Roles of Proton Transfer and Hydrogen Bonding in the Aqueous Electrochemistry of Quinones. *J. Am. Chem. Soc.*, 129, 12847-12856 (2007).

18. Liu, Y., Ye, H.Z., Diederichsen, K.M., Van Voorhis, T., & Hatton, T.A. Electrochemically mediated carbon dioxide separation with quinone chemistry in salt-concentrated aqueous media. *Nat. Commun.*, 11, 2278 (2020).

19. Seo, H. Molecular redox-active organic materials for electrochemical carbon capture. MRS Commun., 13, 994–1008, (2023).

20. Bernstein, M. P., Sandford, S. A., & Allamandola, L. J. The infrared spectra of nitriles and related compounds frozen in Ar and H_2O. *Astrophys. J.*, **476**, 932-942 (1997).

21. Dunwell, M. *et al.*, Examination of near-electrode concentration gradients and kinetic impacts on the electrochemical reduction of CO_2 using surface-enhanced infrared spectroscopy. *ACS Catal.* **8**, 3999-4008 (2018).

22. Xing, Y. et al., Bi@Sn Core–Shell Structure with Compressive Strain Boosts the Electroreduction of CO_2 into Formic Acid. *Adv. Sci.* **7**, 1902989 (2020).

23. Chi, LP. et al., Stabilizing indium sulfide for CO_2 electroreduction to formate at high rate by zinc incorporation. *Nat Commun*. **12**, 5835 (2021).

24. Li, K. et al., In-situ dynamic construction of a copper tin sulfide catalyst for high-performance electrochemical CO_2 conversion to formate. *ACS Catal.* **12**, 9922-9932 (2022).

25. Rabiee, H. et al., Shape-tuned electrodeposition of bismuth-based nanosheets on flow-through hollow fiber gas diffusion electrode for high-efficiency CO_2 reduction to formate. *Appl. Catal., B*, **286**, 119945 (2021).

26. Jiang, X. *et al.*, Oxidation state modulation of bimetallic tin-copper oxide nanotubes for selective CO_2 electroreduction to formate. *Small* **18**, 2204148 (2022).

27. Yao, K. *et al.*, Metal-organic framework derived dual-metal sites for electroreduction of carbon dioxide to HCOOH. *Appl. Catal., B*, **311**, 121377 (2022).

28. Liu, P-X., Peng, L-W., He, R.-N., Li, L.-L. & Qiao, J.-L. A high-performance continuous-flow mea reactor for electroreduction CO_2 to formate. *J. Electrochem.* **28**, 2104231 (2022).

29. Yang, J. *et al.*, Bi-based metal-organic framework derived leafy bismuth nanosheets for carbon dioxide

electroreduction. *Adv. Energy Mater.* **10**, 2001709 (2020).
